# Muscle‐specific gene editing improves molecular and phenotypic defects in a mouse model of myotonic dystrophy type 1

**DOI:** 10.1002/ctm2.70227

**Published:** 2025-02-16

**Authors:** Mariapaola Izzo, Jonathan Battistini, Elisabetta Golini, Christine Voellenkle, Claudia Provenzano, Tiziana Orsini, Georgios Strimpakos, Ferdinando Scavizzi, Marcello Raspa, Denisa Baci, Svetlana Frolova, Spyros Tastsoglou, Germana Zaccagnini, Jose Manuel Garcia‐Manteiga, Genevieve Gourdon, Silvia Mandillo, Beatrice Cardinali, Fabio Martelli, Germana Falcone

**Affiliations:** ^1^ Institute of Biochemistry and Cell Biology CNR Rome Italy; ^2^ Present address: Department of Molecular Medicine Sapienza University of Rome Rome Italy; ^3^ CNR‐EMMA INFRAFRONTIER‐IMPC Rome Italy; ^4^ Molecular Cardiology Laboratory IRCCS Policlinico San Donato Milan Italy; ^5^ Center for Omics Sciences IRCCS Ospedale San Raffaele Milan Italy; ^6^ Sorbonne Université Inserm, Institut de Myologie Centre de Recherche en Myologie Paris France

**Keywords:** CRISPR/Cas9, CTG repeats, DM1, DMPK, DMSXL mouse model, gene editing, muscle, MyoAAV, myotonic dystrophy type 1

## Abstract

**Background:**

Myotonic dystrophy type 1 (DM1) is a genetic multisystemic disease, characterised by pleiotropic symptoms that exhibit notable variability in severity, nature and age of onset. The genetic cause of DM1 is the expansion of unstable CTG‐repeats in the 3′ untranslated region (UTR) of the *DMPK* gene, resulting in the accumulation of toxic CUG‐transcripts that sequester RNA‐binding proteins and form nuclear foci in DM1 affected tissues and, consequently, alter various cellular processes. Therapeutic gene editing for treatment of monogenic diseases is a powerful technology that could in principle remove definitively the disease‐causing genetic defect. The precision and efficiency of the molecular mechanisms are still under investigation in view of a possible use in clinical practice.

**Methods:**

Here, we describe the application of the clustered regularly interspaced short palindromic repeats (CRISPR)/CRISPR‐associated protein 9 (Cas9) strategy to remove the CTG‐expansion in the *DMPK* gene in a mouse model carrying the human transgene from a DM1 patient. To optimise the editing efficiency in vivo, we identified new tools that allowed to improve the expression levels and the activity of the CRISPR/Cas9 machinery. Newly designed guide RNA pairs were tested in DM1‐patient derived cells before in vivo application. Edited cells were analysed to assess the occurrence of off‐target and the accuracy of on‐target genomic events. Gene editing‐dependent and ‐independent mechanisms leading to decreased accumulation of the mutated *DMPK* transcripts were also evaluated.

**Results and Conclusion:**

Systemic delivery of CRISPR/Cas9 components in DM1 mice, through myotropic adeno‐associated viral vectors, led to significant improvement of molecular alterations in the heart and skeletal muscle. Importantly, a persistent increase of body weight, improvement of muscle strength and body composition parameters were observed in treated animals. Accurate evaluation of CRISPR/Cas9‐mediated‐phenotypic recovery in vivo is a crucial preclinical step for the development of a gene therapy for DM1 patients.

**Key points:**

- In vivo application of a therapeutic gene editing strategy for permanent deletion of the pathogenetic CTG‐repeat amplification in the *DMPK* gene that causes myotonic dystrophy type 1.
- Following treatment, diseased mice show a significant improvement of both molecular and phenotypic defects.

## Graphical abstract

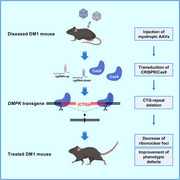

## 1 INTRODUCTION

Myotonic dystrophy type 1 (DM1) is the most common muscular dystrophy in adults characterised by pleiotropic symptoms, which are highly variable in their nature and severity.^1^, ^2^ Major muscular features include myotonia, muscle weakness, atrophy and smooth muscle dysfunction. Cardiac conduction defects and arrhythmias are associated with cardiomyopathy and may lead to sudden death.^1^, ^2^ In the brain, predominant structural abnormalities of the white matter are observed, as well as cognitive impairment (such as executive dysfunction, visuospatial deficits and abnormal social cognition), behavioural changes (such as apathy and social avoidance) and excessive daytime sleepiness.^3^, ^4^ Additionally, body composition abnormalities including increase in fat mass correlated with muscle weakness are reported in DM1 patients.^5^, ^6^ DM1 is caused by an expanded (CTG)n repeat in the 3′ untranslated region of the dystrophia myotonica protein kinase (*DMPK*) gene on chromosome 19.^7^, ^8^ The severity of the disease and the age of onset are correlated with the number of CTG repeats: a ‘mild’ phenotype is associated with 50 to 150 repeats, a ‘classic’ phenotype with 100 to 1000 repeats, and a ‘congenital’ phenotype with more than 1000 repeats. In unaffected individuals, the number of repeats is typically fewer than 50.^9^ A similar molecular alteration is observed in myotonic dystrophy type 2, resulting from an unstable tetranucleotide repeat expansion, CCTG, in intron 1 of the nucleic acid‐binding protein *CNBP* gene on chromosome 3q21.^10^ Although different pathogenic mechanisms may contribute to DM1 disease,^1^, ^11^, ^12^ large experimental evidence converges in assigning the main toxic function to the expanded *DMPK* transcripts that sequester members of the muscleblind‐like (MBNL) family of RNA‐binding proteins in cell nuclei forming typical nuclear foci in cells of DM1 patients. Nuclear retention of MBNL proteins and stabilisation of the CUGBP Elav‐like family member 1 protein leads to aberrant splicing and alternative polyadenylation of many transcripts, as well as abnormal microRNA processing, mRNA dysregulation and up‐regulation of circular RNA expression.^13^, ^14^, ^15^, ^16^, ^17^ Although DM1 is primarily driven by toxic RNA gain‐of‐function mechanisms, epigenetic alterations, such as hypermethylation and increased repressive histone modifications, are present at the mutated *DMPK* locus.^18^, ^19^, ^20^, ^21^ These alterations have been associated to variations in muscle strength and respiratory traits,^22^ and may contribute to the down‐regulation of the nearby *SIX5* gene.^23^, ^24^

The understanding of the pathogenetic mechanisms of the disease was facilitated by the availability of both in vitro and in vivo models.^25^ Established cultures of cells derived from DM1 patients or engineered cell lines with CTG repeats of different length were shown to reproduce the DM1 hallmarks.^26^, ^27^, ^28^, ^29^, ^30^ For in vivo studies, transgenic mouse models expressing the mutant *DMPK* gene or synthetic CTG‐repeats were established and characterised for their similarity to the human disease.^25^, ^26^, ^27^, ^28^, ^29^, ^30^, ^31^ Among the available mouse models, the DMSXL transgenic model carries a 45 kb portion of the human DM1 locus including a *DMPK* gene with >1000 CTG repeats and the adjacent upstream *DMWD* gene and downstream *SIX5* gene from a DM1 patient.^32^ This model has been used for testing both drug‐mediated therapies and gene‐editing approaches in a human genetic context.^33^, ^34^, ^35^, ^36^ In homozygous DMSXL mice, the ubiquitous expression of expanded *DMPK* transcripts from the human DM1 locus results in multisystemic phenotypes reproducing molecular alterations as well as many aspects of the human pathology including reduced muscle strength, lower motor performances, peripheral neuropathy, cardiac conduction defects, cognitive and behavioural abnormalities.^32^, ^37^, ^38^, ^39^, ^40^, ^41^ These characteristics make this model particularly suitable for testing the effects of therapeutic treatments on DM1‐related phenotypic alterations, with results that can be more easily translated to the human context. Both in vitro and in vivo models have been very useful to discover crucial molecules and cellular pathways involved in the pathology and to test therapeutic approaches.^25^ Despite the encouraging results obtained in preclinical studies using therapeutic strategies aimed at targeting the mutant allele or downstream signalling pathways, these approaches have shown some important limitations, such as the need for repeated administration, lack of sufficient specificity, poor cellular uptake and, more importantly, their failure to eradicate the disease‐causing mutation.^25^

The recent application of the prokaryotic CRISPR (clustered regularly interspaced short palindromic repeats)/Cas9 (CRISPR‐associated protein 9) system for genome editing has provided a flexible technology exploiting the cleaving capacity of Cas9 which, binding to engineered single guide RNAs (sgRNAs), can reach specific DNA or RNA sequences in virtually any genomic location.^42^, ^43^, ^44^ Different editing strategies employing Cas9 or TALEN nucleases have been used to interfere with accumulation of the toxic *DMPK* transcript in DM1 cell nuclei.^25^ Deactivated Cas9 and CRISPR interference system were used to block *DMPK* gene transcription,^45^, ^46^ RNA‐targeting Cas9 to degrade CUG‐repeated RNA,^47^ insertion of a premature polyA signal in the *DMPK* gene was used to stop transcription upstream of the mutated *DMPK‐*3′UTR region.^48^ All these treatments led to rescue of splicing defects and reduction of nuclear foci in vitro and/or in vivo.^45^, ^47^, ^49^ However, they either require continuous expression of the CRISPR elements or do not eliminate the pathogenic CTG‐expansion at the *DMPK* locus.^12^, ^20^, ^23^, ^50^ CRISPR/Cas9‐mediated technology was also applied to excise permanently the DM1 expansion by simultaneous double strand breaks (DSBs) in the regions upstream and downstream the CTG‐repeats. Successful removal of the repeats was obtained in many different cell models and resulted in reduction of nuclear foci and improvement of aberrant splicing.^28^, ^34^, ^48^, ^49^, ^51^ In vivo injection of adeno‐associated viruses (AAVs) expressing CRISPR/Cas9 using the *Staphylococcus aureus* Cas9 in skeletal muscle of DMSXL mice led to repeat deletion and a decrease in ribonuclear foci.^35^ More recently, we have obtained time‐restricted and muscle‐specific CTG‐repeat deletion in DM1 patient‐derived myogenic cells and in skeletal muscle of DMSXL mice using a tetracycline‐repressor‐based CRISPR/enhanced *Streptococcus pyogenes* Cas9 (eSpCas9) system.^36^

In view of a gene therapy application in DM1 patients, specificity, safety and efficacy of gene editing need to be carefully evaluated. Previous work from our and other research groups have addressed this issue in cells derived from DM1 patients and, in most cases, no off‐target effects were observed.^28^, ^51^, ^52^ Unintended on‐target effects such as large deletions, inversions or base substitutions, however, were occasionally observed.^53^, ^54^, ^55^, ^56^ In myogenic cells, we found that the occurrence of these events correlated with the cell‐cycle state of the target cells, being more frequent in proliferating rather than in differentiated cells.^36^ A way to control editing activity and reduce undesirable effects is to target the expression of the CRISPR/Cas9 complex to specific cells by using suitable promoters combined with recently developed tissue‐specific transducing vectors.^57^, ^58^ Here, we describe a gene editing application for deletion of the CTG‐repeat expansion in the human *DMPK* gene both in cells derived from a DM1 patient and in DMSXL mice, using novel tools selected for high editing efficiency and specificity. Transduction of these CRISPR/Cas9 components in vivo via myotropic AAVs led to effective gene editing and improvement of both molecular and phenotypic alterations in the injected mice.

## 2 METHODS

### 2.1 Lentiviral and adeno‐associated viral constructs

Plasmids used for production of Lenti–CK8–Cas9 (Figure S1A) and single strand (ss) AAV–CK8–Cas9 (Figure S1B) were previously described.^36^ pLenti–CK8–dCas9 (Figure S1A) was generated by replacing eSpCas9 (1.1) with dead/inactive eSpCas9 (1.1) derived from pX330‐Flag‐deSpCas9 (Addgene #92114; Cambridge, MA, USA). pLenti–H1–sgC3/384 (Figure S1A) was derived from pLenti–H1–Tet–sgRNA–mCherry^36^ by replacing the sg34/589 cassette with the in vitro synthesised sgC3/384 cassette (GenScript, Piscataway, NJ, USA). pLenti–H1–sgC3 and pLenti–H1–sg384 were derived from pLenti–H1–sgC3/384 by restriction digestion. For in vivo application, sg34/589 pair^36^ and sgC3/384 pair were placed under the control of the human U6 promoter and cloned into self‐complementary adeno‐associated vectors (scAAVs)^59^ (Figure S1B). Due to packaging size constraints, scAAVs were used only for sgRNAs, while single strand AAV vectors (ssAAV) were employed for eSpCas9 under the control of the muscle‐specific promoter CK8^36^ (Figure S1B). The capsid variant coding plasmid for production of myotropic AAVs was generated by insertion of the MyoAAV 2A variant‐specific sequence ‘gggccgggtagaggagaccagactacgttg’^57^ within the pAAV2/9n vector (Addgene #112865) capsid sequence.

### 2.2 Cell culture, ribonucleoprotein transfection and lentiviral infection

Myogenic cell lines were obtained from primary dermal fibroblasts of a DM1 patient, carrying 520 CTG amplifications in the 3′UTR of the *DMPK* gene (DM1 cells), and from an unaffected individual (control cells – CT). Both cell lines were transduced with hTERT and oestrogen‐inducible MYOD1, as previously described.^28^ Due to CTG repeat instability, during in vitro culture the number of triplets further increased to >1000, as estimated by Northern blot analysis of the mutated transcript.^28^ Myogenic cells were propagated in growth medium (GM), consisting of Dulbecco's modified Eagle's medium (DMEM) without phenol red (Gibco, Thermo Fisher Scientific, Waltham, MA, USA) supplemented with 15% foetal bovine serum (Gibco, Thermo Fisher Scientific). Cells grown to confluency on dishes coated with 50 µg/mL Collagen I (Gibco, Thermo Fisher Scientific) were induced to differentiate to myotubes with differentiation medium (DM), consisting of DMEM without phenol red supplemented with 10 µg/mL insulin (Sigma–Aldrich, St. Louis, MO, USA), 10 µg/mL transferrin (Gibco, Thermo Fisher Scientific) and 10^−7^ M β‐oestradiol (Sigma–Aldrich). The differentiation ability of the cells used in this study was previously described.^28^ For fluorescent in situ hybridisation (FISH) and RNA analysis, cells grown in doxycycline (DOX)‐containing GM were incubated in DOX‐containing DM for the last 24 h, since *DMPK* gene transcription increases upon induction of myogenic differentiation^60^ and alternatively spliced isoforms show the most pronounced difference at early differentiation time.^36^ Cells were incubated under a 5% CO2 atmosphere at 37°C. Cell lines tested negative for mycoplasma.

In vitro transcribed sgRNAs (Precision gRNA Synthesis Kit, Invitrogen, Thermo Fisher Scientific) were complexed with Cas9 protein (Sigma–Aldrich) or dead‐Cas9 (dCas9) protein (Sigma–Aldrich) and transfected using Lipofectamine CRISPRMAX Cas9 Transfection Reagent (Invitrogen, Thermo Fisher Scientific), following the manufacturer's instructions. The sequences of the used sgRNAs are listed in Table S1.

Lentiviral particles were produced in 293FT cell line (Thermo Fisher Scientific), as previously described.^61^ DM1 myogenic cells were co‐infected with lentiviral particles carrying Cas9 or dCas9, lacking nuclease activity, and inducible sgRNAs at a 1:1 ratio and a multiplicity of infection of 5 in order to ensure double infection of most cells. Transduced cells co‐expressing EGFP and mCherry were sorted using a Becton Dickinson (BD) FACSAria II or BD FACSDiva 9.4 cell sorter (Flow Cytometry facility, EMBL, Monterotondo, Italy).

### 2.3 Off‐target analysis

Potential off‐targets were identified via querying five publicly available software tools: CRISPRoff (v1.2beta),^62^, ^63^ COSMID,^64^ CRISTA,^65^ Cas‐OFFinder^66^ and ChopChop (version 3).^67^ The resulting lists for each sgRNA were cross‐referenced and ranked in descending order based on the number of overlaps. Specific primers with standard Illumina adapters for library preparation were designed to cover the relevant genomic regions (Table S2A). For each sample, starting from 10 ng of input gDNA, we performed polymerase chain reaction (PCR) using Platinum Taq DNA Polymerase kit (Invitrogen®, Thermo Fisher Scientific), at the following conditions: hold 94°C/2 min, then 35 cycles (94°C/30 s, 62°C/30 s, 72°C/1 min). Among the top 25 of each intersection, shared by at least two tools, 21 and 22 potential off‐target sites for sgC3 and sg384, respectively, were successfully amplified by PCR (Table S2A).

### 2.4 Library preparation for amplicon deep sequencing

Amplicons were purified by AMPURE beads (Beckman Coulter, Brea, CA, USA), with 1:1.2 vol/vol ratio, and resuspended in 30 µL of ddH_2_O. Library preparation and barcoding were performed using Nextera Unique Dual Index (UDI) (Illumina, San Diego, CA, USA) primers, according to the manufacturer's guidelines. PCR amplification was performed using Kapa HiFi polymerase (Roche, Sigma–Aldrich) adopting the following conditions: Hold 98°C/3 min, followed by 18 cycles (98°C/30 s, 55°C/30 s,72°C/1 min) and elongation (72°C for 3 min). PCR products were then purified with AMPURE beads (Beckman Coulter) using 1:0.8 vol/vol ratio. To quantify and verify the integrity of the libraries, a HS1000 DNA Chip on a Tapestation 4100 (Agilent, Santa Clara, CA, USA) was used. All libraries were pooled in equimolar ratios and paired end sequencing with 2 × 250 nt read length and 30% PhiX was performed on a MiSeq 600 v3 (Illumina).

### 2.5 Off‐target variant detection by amplicon deep sequencing

Briefly, the obtained pair‐end reads (250 bp) were trimmed to remove adapters and aligned to hg38 reference genome with BWA‐MEM algorithm.^68^ Subsequently, Freebayes software^69^ was parameterised to identify single‐nucleotide variants (SNVs) and small indels and their frequencies within the predicted off‐target sequence, using parameter‐min‐alternate‐fraction 0.002. Pre‐processing of reads was made using GATK best practices (Genome Analysis Toolkit gatk (v3.7)).^70^ A minimal filter was applied using SnpSift, to retain confidently called variants, requiring (i) variant quality > 0, (ii) variant quality/alternate‐allele‐reads > 1, (iii) at least 1 read from the one strand and 1 from the other to support the alternative allele, (iv) more than 1 read supporting both flanks (upstream and downstream of the variant site) and (v) a mean mapping quality of at least 10 for the reads supporting the reference or the alternative allele. Amplicons exhibiting a mean coverage below 10 across samples were excluded from further investigations.

### 2.6 Cas9‐mediated target enrichment for Oxford Nanopore Technology sequencing

#### 2.6.1 Probe design

For the Oxford Nanopore Technology (ONT) ‘Cas9 excision approach’,^71^ CRISPR RNAs (crRNAs) were designed to cut the region of interest (ROI) four times, that is, twice upstream on the (+) strand and twice downstream on the (−) strand, to avoid incomplete cleavage. A custom tool developed by OHMX.bio (Ghent, Belgium) was used to design all probes. This tool combines CHOPCHOP (version 3) and IDT primer design tools (Integrated DNA Technologies [IDT], Coralville, IA, USA) with a dbSNP check and a BLAST and BLAT homology search. A total of 12 crRNA probes (*S. pyogenes* Cas9 Alt‐R™ crRNAs; IDT) were screened in a multiplexed manner to enrich two different ROIs: one encompassing the full‐length *DMPK* gene (∼14 kbp) to screen for potentially occurring long deletions, the other flanking the sgC3/sg384 editing sites located in the 3′UTR region (∼4 kbp). The crRNA pool yielding the highest target coverage on the tested human genomic DNA reference sample (human male; Promega, Madison, WI, USA), was selected for subsequent sequencing (Table S3A). For both chosen cRNA‐pools, the rate of reads mapping to the ROI was 0.2% of all ONT‐sequenced reads. For Cas9 enrichment of the full‐length *DMPK* region, we used one edited DM1 myogenic cell population in a differentiated cell state. For the shorter 3′UTR region, two cell populations of each cell state, proliferating (GM) and differentiated (DM), were processed.

#### 2.6.2 Extraction of high‐molecular‐weight DNA

Genomic DNA dedicated to targeted ONT sequencing was isolated using the MagAttract HMW DNA kit (Qiagen, Venlo, Netherlands), to preserve high‐molecular‐weight DNA (100–200 kb). Where necessary, sample concentration was performed with Agencourt AMPure XP beads (Beckman Coulter) following ONT's protocol ‘SPRI size selection protocol for fragments > 1.5–2 kb’. DNA concentration was determined with the Qubit dsDNA HS Assay Kit (Life Technologies, Thermo Fisher Scientific). DNA integrity was verified on the Agilent Tapestation 4200 (Agilent).

#### 2.6.3 Target enrichment and library preparation

All steps were carried out following the Nanopore Cas9 targeted sequencing protocol. Briefly, for each pool, equal volumes of the four selected crRNA probes (*S. pyogenes* Cas9 Alt‐R™ crRNAs; IDT) were combined. Equimolar amounts of crRNA pools and tracrRNA were annealed. Cas9 ribonucleoprotein complexes (RNPs) were formed by assembling the previously annealed crRNA–tracrRNA pools with HiFi Cas9 (Alt‐R® *S. pyogenes* HiFi Cas9 nuclease V3; IDT). Input gDNA (3.3–7 µg) was first dephosphorylated to reduce non‐specific adapter‐ligation, subsequently cleaved with Cas9 RNPs and A‐tailed using ATPs and Taq polymerase. AMX adapters (Ligation Sequencing kit; Oxford Nanopore Technologies, Oxford, UK) were ligated to Cas9 cut sites and the resulting libraries were then purified and concentrated using AMPure XP beads (Beckman Coulter).

#### 2.6.4 Sequencing

R9.4.1 MinION flow cells (Oxford Nanopore Technologies) were first primed and then loaded with the processed libraries, according to manufacturer's instructions. 24 h of sequencing run were performed on the GridION (Oxford Nanopore Technologies). During sequencing, real‐time basecalling was enabled using the high‐accuracy model of Guppy (v6.5.7).

#### 2.6.5 Bioinformatic analysis of ONT sequencing

Initially, read quality was assessed using PycoQC (2.5.2), applying a filter with a Q score of 9. All passing reads were then concatenated per sample into a single FASTQ file. Subsequently, Minimap2 (v 2.24, default settings for ONT) was employed for read alignment, against the human reference (GRCh38) and reference sequences of non‐edited mutated allele, CTG‐repeats excision and inversion. For construction of the mutated allele reference, an additional 1600 triplets (4800 nt) were inserted within the repeat region, guided by the observed reads. The CTG‐repeats excision reference represents the sequence expected for simultaneous, precise sgC3/384 cut followed by repeat removal (double‐cut) and perfect re‐joining. The inversion reference is the sequence predicted for sgC3/384 cut followed by reinsertion in the reverse orientation. Samtools (v1.6) was employed to subset the reads aligned to the ROI, sort and index the alignment files into sorted BAM files. Subsequently, IGV (v 2.16.2) was used for the manual examination of all primary reads. We assessed alignments against the human reference (GRCh38) for the investigation of non‐edited wild‐type (WT) alleles and sgC3/384 target regions in the absence of CTG excision. For analysis of non‐edited mutated alleles, CTG‐repeats excision and inversions, we examined the corresponding alignments. To determine the read count in each category, we specifically considered only reads spanning a region extending at least 5 base pairs upstream and 5 base pairs downstream of the predicted cleavage sites of sgC3 and sg384, respectively. Editing accuracy was evaluated by investigating 5 bp up‐ and 5 bp downstream of the predicted cut and repair sites expected for either single cut by sgC3 and sg384, or the double‐cut.

### 2.7 Animal experiments and AAV injections

The DMSXL mouse strain (>90% C57BL/6 background) carries 45 kb of human *DMPK* genomic region cloned from a DM1 patient as previously described.^32^, ^72^ A DMSXL mouse colony was established by crossing hemizygous male and female mice. Triplet expansion was verified in each hemizygous mouse before mating by long PCR and Southern blot.^28^ Progeny was genotyped by PCR as described.^73^ Homozygous DMSXL mice show a high perinatal mortality, are underweight and require special care after weaning.^32^ For optimal feeding, gel diet (DietGel76A; Clear H20, Westbrook, ME, USA) and wet food (EMMA23 protein‐enriched diet; Mucedola, Settimo Milanese, Italy) were provided on the floor of the cage. High‐titre stocks of MyoAAVs were produced by InnovaVector (Pozzuoli, Italy). The intraperitoneal (IP) injections were performed in homozygous male and female DMSXL mice at post‐natal day 5 and 24 (P5 and P24). Mice at P5 were injected with a volume of 50 µL containing 2.2–5 × 10^11^ vg of each vector (nuclease and targeting), while mice at P24 with a volume of 175 µL containing 5.6–15 × 10^11^ vg of each vector. The highest doses of AAVs in the maximum injectable volumes at each age were used. Untreated and PBS‐injected mice were used as control. Having verified that there were no measurable differences (in mortality rate, body weight and in behavioural tests) between untreated and PBS‐treated mice, they have all been included in the same experimental group and defined as not treated (NT). At 4 weeks post‐injection, mice were sacrificed and tibialis anterior (TA), gastrocnemius, diaphragm, heart and liver tissues were collected and snap frozen in liquid nitrogen for DNA and RNA extraction, or were immediately included in frozen Tissue‐Tek OCT Compound (Sakura Finetek, Venice, IT) for histological analysis.

### 2.8 Genomic DNA analysis

Genomic DNA was isolated from cultured cells using the Kapa Express Extract kit (Roche, Sigma–Aldrich) or the DNeasy Blood & Tissue Kit (Qiagen). Genomic DNA was extracted from mouse tissues by overnight digestion in proteinase K buffer (100 mM Tris–HCl [pH 8.5], 5 mM EDTA [pH 8], 0.2% SDS, 200 mM NaCl and 0.5 mg/mL proteinase K) at 55°C in a thermal mixer (Biosan, Riga, Latvia) with agitation at 1400 rpm. Samples were treated with RNAse A at a concentration of 0.5 mg/mL and extracted using phenol‐chloroform. Genome editing was evaluated by standard PCR using KAPA2G Fast HS Genotyping Mix (2×) (Roche, Sigma–Aldrich) on approximately 10 ng of genomic DNA from DM1 cells and 60 ng of genomic DNA from mouse tissues and amplifying with DMPK F2–DMPK R2 primers (Table S1) for 40 cycles.

The number of CTG‐deleted molecules was evaluated by absolute qPCR using the standard curve method. A standard curve was set up using a PCR‐amplified fragment containing the Cas9‐34/589 CTG‐deleted region; the obtained DNA was accurately quantified by measuring OD at A260 and the number of copies was calculated according to its molecular weight. The purified standard DNA was diluted over several orders of magnitude covering the expected quantity range of the samples to be analysed. The standard curve DNA and the genomic DNA from the Cas9‐34/589 treated samples were amplified to quantify *DMPK* deleted copies (34/589 del F–34/589 del R) along with total *DMPK* copies (DMPK ref1 F–DMPK ref1 R). The number of Cas9‐34/589 *DMPK* deleted molecules was normalised to the number of total *DMPK* molecules and expressed in percentage.

### 2.9 RNA FISH and immunofluorescence analysis

Cells were fixed with 2% formaldehyde and subjected to FISH using a (CAG)6CA probe labelled with Texas red at the 5′end (IDT) as described previously.^74^ Nuclei were visualised with Hoechst 33258 dye. Frozen muscles were cut in 8 µm‐sections and stored at −80°C. For FISH analysis, the cryosections were thawed at RT for 30 min and then fixed with 2% PFA in PBS. After permeabilisation with pre‐chilled 2% acetone, slides were prehybridised in 40% formamide, 2× SSC buffer for 10 min at RT and then incubated in hybridisation buffer (2× SSC, 40% formamide, 0.02% BSA, 2 mM vanadyl ribonucleoside complex, 67 mg/mL tRNA) containing 1 ng/mL of (CAG)_6_CA probe for 2 h at 37°C. After washes, nuclei were counterstained with Hoechst 33258 dye.

Cell slides were examined with an Olympus AX70 immunofluorescence microscope. Images were captured with an Olympus XM10 camera and processed using Olympus CellSens Standard software, version 1.8.1. Images of heart and TA tissue sections were acquired using an Olympus FV1200 confocal microscope (Olympus, Tokyo, Japan) at 60X magnification and visualised with FV10‐ASW software (version 4.2; Olympus). The counting of foci numbers was performed blindly using ImageJ software. A minimum of 200 nuclei per sample were counted in cultured cells, while at least 500 nuclei for each individual were counted in tissue sections. In cultured cells, where foci number can be very high (>20), different foci number ranges were evaluated, while only foci‐positive or ‐negative nuclei were counted in tissue sections. For cultured cells, the average percentage of foci from different experiments is shown. For tissue sections, due to the higher variability of FISH staining in mouse tissues compared with cell cultures, data were expressed as average fold change of foci‐positive nuclei in treated mice relative to foci‐positive nuclei in untreated mice taken as 1.

### 2.10 RNA analysis

Total RNA was extracted with TRIzol reagent (Invitrogen, Thermo Fisher Scientific) following the manufacturer's instructions. Mouse tissues were minced into small pieces and disrupted by Tissue Lyser (Qiagen), and cultured cells were lysed on plates. RNA samples were then treated with gDNAEraser (Takara Bio, Kusatsu, Japan) to eliminate traces of DNA, and reverse‐transcription was performed with the PrimeScript RT‐reagent kit (Takara Bio) using oligo(dT) and random primers. Primers used for qRT‐PCR analysis are listed in Table S1. PowerUP SYBR Green PCR Master Mix (Applied Biosystems, Thermo Fisher Scientific) was used to analyse RNAs by qPCR using either a 7500 or a 7900HT Fast Real‐Time PCR System (Applied Biosystem, Thermo Fisher Scientific). Relative expression was calculated either with the standard curve method or the comparative Ct method using the reference genes indicated in the figure legends.

For alternative splicing (AS), analysis was performed using primers specific for each alternative isoform normalised to the total amount of transcript, as described in Ref. ^36^. To establish the % rescue due to CRISPR/Cas9 activity the following equation was used^75^ % rescue = [(DM1 EI − DM1_treated EI)/(DM1 EI − CT EI)] × 100, where DM1 EI = % exon inclusion in DM1, DM1_treated EI = % exon inclusion in DM1 CRISPR/Cas9 treated samples, CT EI = % exon inclusion in control samples.

Polyadenylated mRNA was isolated from myogenic cells, grown in GM, and induced to differentiate in DM for 24 h, using the GeneElute Direct mRNA Miniprep kit (Sigma–Aldrich), following the manufacturer's instructions. For dot‐blot analysis, denatured total RNA (100–2000 ng/dot) or polyA^+^ mRNA (50–100 ng/dot) was loaded on a dot‐blot apparatus (Schleicher & Schuell GmbH, Dassel, Germany) under vacuum conditions. *DMPK* transcript analysis by dot‐blot could be performed in mouse heart tissues only when sufficient amount of RNA was available. RNA was transferred onto positively charged nylon membranes (Roche, Sigma–Aldrich) and hybridised with DIG‐labelled (CAG)_6_ LNA‐oligonucleotide (Eurogentec, Liège, Belgium) and with a probe to human *GAPDH* (NM_002046.7, nt 21–413) or mouse *Gapdh* (NM_001411840.1, nt 246–653), both generated with DIG‐High Prime DNA Labeling Kit (Roche, Sigma–Aldrich). The probe‐target hybrids were visualised through a chemiluminescent assay using the CDP‐Star substrate (Roche, Sigma–Aldrich) and the ChemiDoc XRS+ Imaging System (Bio‐Rad, Hercules, CA). RNA dot blots were quantified using Image Lab 6.1 software (Bio‐Rad).

### 2.11 Body weight, grip strength and body composition assessment

DMSXL homozygous mice injected at P5 were weighed weekly, for some animals starting at age 1 week. Most of them were then sacrificed at 5 weeks of age for tissue analysis. A small experimental group was monitored up to 16 weeks of age. Not all mice were weighed at each time point (Table S4). DMSXL homozygous mice injected at P24 were also weighed every week after injection and all sacrificed at 8 weeks of age for tissue analysis. All DMSXL homozygous mice weighed in our facility from the beginning of the study were included in the NT group and used as reference.

Grip strength test was performed as described^76^, ^77^ at age 5 or 8 weeks. Briefly, mice were gently pulled by their tail while gripping with the front or all four paws to a small grid attached to a dynamometer (Bioseb, France). Peak force in grams was recorded over three consecutive trials with 1 min of inter‐trial interval. Median values were considered for the analysis.

Bone health and body composition were comprehensively evaluated at the age of 4 months using Dual Energy X‐ray Absorptiometry (DEXA) analyser (The PARAMETER™ XPERT® 40 Cabinet X‐ray System, T00261_A 9.2019; Kubtec Scientific, 111 Research Drive Stratford, CT 06615, USA). Mice were sacrificed by CO_2_ inhalation and the body length was also measured along a ruler. The procedure was performed according to the open‐access International Mouse Phenotyping Consortium (IMPC) standard operating procedures (https://www.mousephenotype.org/impress/ProcedureInfo?action=list&procID=548). In the DEXA analysis, the head region was excluded from the whole‐body area of interest. The software then automatically calculated various body composition parameters from the remaining image, including total body bone mineral content (BMC in grams), bone mineral density (BMD in grams/cm^2^), fat and lean mass (grams), fat and lean mass percentage (%). Adiposity index was calculated as the ratio between fat and lean mass.

### 2.12 Statistical analysis

StatView 5.0, GraphPad Prism 8.3.1 (GraphPad Software, San Diego, CA) software were used for statistical analysis. Continuous variables were analysed by Student's *t*‐test, Welch's *t*‐test, Mann–Whitney test, one‐way ANOVA with Fisher's PLSD or Tukey's post‐hoc tests or simple linear regression (slopes comparison). To assess the difference in weight gain over time between NT and MyoAAV–C3/384‐treated DMSXL mice, separate linear mixed effects models for each sex were applied using the *lmerTest* R package.^78^ The models included the interaction between treatment and time as a fixed effect and individual mouse IDs as a random effect to account for subject‐specific variability. Survival analysis was performed with Kaplan–Meier curves, assessing difference via Log‐rank (Mantel–Cox) test. Significance level was set at *p* < .05. Continuous variables were expressed as mean ± standard error of the mean (SEM) with error bars representing the standard error of the mean.

## 3 RESULTS

### 3.1 Selection of sgRNA pairs

Before proceeding with the systemic delivery of the CRISPR/Cas9 system in vivo, we sought to optimise gene editing efficacy starting from the selection of the most effective sgRNA pair to delete the pathogenic CTG‐repeat expansion. To this aim, we employed the previously described sgRNAs (34, 384 and 589)^28^ and designed new sgRNAs upstream (C1, C3) and downstream (C2, CH1, LS23) the CTG‐repeats by using dedicated algorithms (https://crispeta.crg.eu/; https://chopchop.cbu.uib.no/) or manually (Figure S2A and Table S1). The choice of sgRNA target sequences is limited by the size of *DMPK* 3′UTR and by the need of SpCas9‐specific PAM sequences. Different combinations of in vitro transcribed sgRNAs were transfected along with SpCas9 protein in the previously described immortalised fibroblast line derived from a DM1 patient.^28^, ^36^ Next, editing efficiency was tested by PCR, along with the disappearance of ribonuclear foci containing CUG‐repeats measured through FISH with a fluorescent (CAG)_6_CA probe (Figure S2B). Based on these results and on the proximity of the sgRNAs to the repeat region, the pair sgC3/384 was selected for the subsequent experiments.

### 3.2 Functional characterisation of the new sgC3/384 pair

**FIGURE 1:**
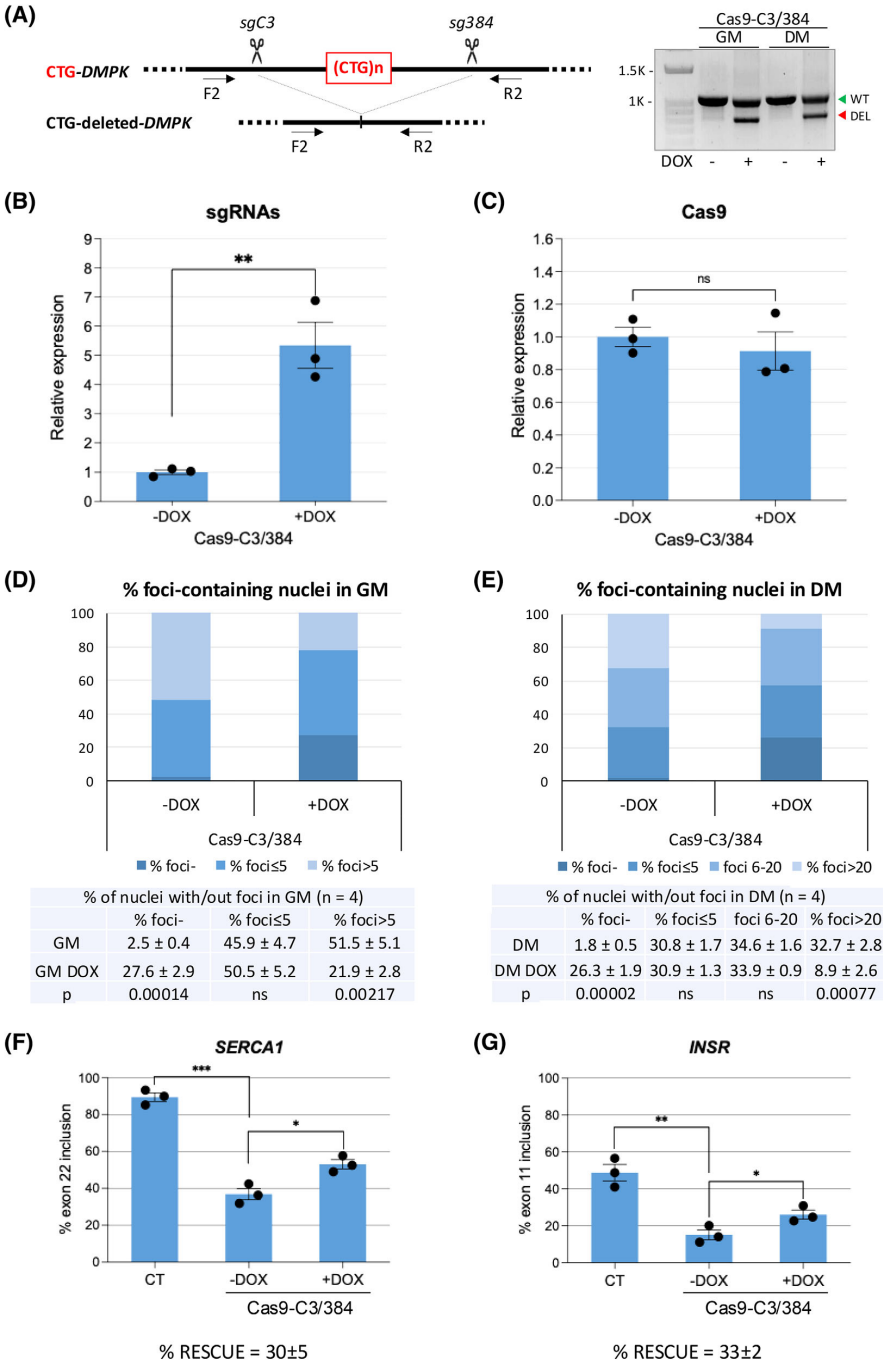
Characterisation of the sgC3/384 pair. DM1 cells transduced with lentiviruses expressing Cas9 and DOX inducible sgC3/384 were maintained in growth medium (GM) or induced to differentiate into myotubes in differentiation medium (DM) in the absence or in the presence of DOX. (A) *DMPK* gene editing was induced upon administration of DOX for 5 days in GM or in DM. Genomic DNA was extracted and analysed by PCR using *DMPK* primers binding upstream (F2) and downstream (R2) of CTG expansion, as shown in the diagram. Black scissors indicate the sgRNA cutting sites. The green triangle indicates undeleted WT allele‐derived amplicons, the red triangle indicates the expected CTG‐deleted products (DEL) from both wild‐type and mutated alleles. (B and C) qRT‐PCR of sgRNAs and Cas9 mRNA expressed in cells maintained in GM with or without DOX for 5 days. The primer pair used for sgRNA analysis can amplify both sgRNAs. Expression of sgRNAs and Cas9 was normalised on *RPL23* mRNA and expressed relative to the levels measured in untreated cells set as 1 (mean ± SEM). *n* = 3; **p = .0053; ns = not significant. (D and E) RNA FISH analysis of DM1 cells transduced as described, following DOX treatment for 5 days. Histograms show the percentage of nuclei containing no foci (foci‐), ≤5 foci, and >5 foci in growing cells (GM, D) and no foci (foci‐), ≤5 foci, between 6 and 20 foci (foci 6–20), and >20 foci in differentiated cells (DM, E). The tables below the graphs present the mean percentages, standard errors, and *p* values. *n* = 4; ns = not significant. (F and G) qRT‐PCR analysis of *SERCA1* and *INSR* transcripts in cells untreated or treated with DOX for 5 days in GM was performed using primers specific for each alternative isoform normalised to the total amount of *SERCA1* and *INSR* transcripts, respectively. Histograms represent the mean percentage of exon inclusion in the absence or presence of DOX, compared with the control cells (CT) obtained from individuals not affected by DM1 (mean ± SEM). *n* = 3; *p < .0150 (F), *p < .0387 (G), **p < .0029, ***p < .0002. The percentage of rescue is indicated. Each dot in the graphs represents an individual sample from independent experiments. The statistical analyses presented in the figure were conducted using an unpaired *t*‐test, with Welch's correction applied where appropriate.

To study the editing efficiency and specificity of the new sgC3/384 pair along with eSpCas9 in stable conditions, we infected myoblast‐convertible immortalised DM1 fibroblasts with Lenti–CK8–Cas9 and Lenti–H1–sgC3/384. Co‐infected fibroblasts were sorted and maintained in GM or induced to differentiate into myotubes by activation of oestrogen‐inducible MYOD1 in DM. Expression of sgRNAs upon administration of DOX for 5 days led to inducible deletion of CTG repeats in the 3′UTR of the *DMPK* gene, while Cas9 was constitutively expressed (Figure 1A–C). The effect of CRISPR/Cas9 gene editing on reduction/elimination of ribonuclear foci containing CUG repeats was evaluated through FISH. Due to the increased transcription and stability of *DMPK* transcript upon induction of myogenic differentiation,^60^ the number of foci per nucleus is usually much higher in myotubes than in fibroblasts or undifferentiated cells. In DOX‐treated cells the number of foci was strongly reduced both in proliferating cells in GM and in differentiated cells in DM (Figure 1D,E). Notably, around 27% of nuclei were completely foci‐free in both conditions, compared with less than 3% in untreated cells, and the number of foci in the remaining nuclei was strongly reduced. To verify whether the decrease of ribonuclear foci was accompanied by a reduction in the mutated *DMPK* transcript, RNA dot‐blot analysis was performed using a (CAG)_6_‐probe that allows to monitor expanded CUG levels in DM1 cells compared with CT(Figure S3A). As expected, a significant reduction (about 30%) in the accumulation of the mutated *DMPK* transcript was observed following CRISPR/Cas9 treatment (Figure S3B–E). In contrast, total *DMPK* mRNA levels remained unaffected by Cas9–C3/384 treatment (Figure S3F,G). Given the proximity of *SIX5* gene to the edited region of *DMPK* (Figure S2A), we evaluated whether *DMPK* gene editing could affect its expression in untreated and DOX‐treated DM1 cells, compared with CT. Consistent with previous findings,^23^, ^24^ a reduction in *SIX5* mRNA expression was observed in DM1 cells carrying the CTG expansion. Following DOX induction, *SIX5* expression in edited DM1 cells increased significantly to levels comparable to those of CT (Figure S4A). Next, AS of sarcoplasmic/ER calcium ATPase 1 (*SERCA1*) and insulin receptor (*INSR*) transcripts, previously found to be defective for exon inclusion in DM1 myogenic cells,^25^ was analysed by qRT‐PCR in untreated and DOX‐treated DM1 cells, compared with CT. Both *SERCA1* and *INSR* normal transcript forms, containing exon 22 and exon 11, respectively, showed an increase following DOX induction, corresponding to a rescue of 30 and 33%, respectively (Figure 1F,G). Taken together these results suggest that the new sgRNA pair is effective in reducing the main DM1‐related molecular hallmarks in patient‐derived cells.

### 3.3 Off‐target analysis

To evaluate potential off‐target interactions of sgC3 and sg384, we took advantage of five established prediction algorithms and potential off‐targets shared by at least two tools were selected for validation by amplicon‐sequencing. Specifically, 21 and 22 potential off‐target sites for sgC3 and sg384, respectively, were successfully amplified by PCR (Table S2A). Amplicons were generated from genomic DNA of edited proliferating (GM Edited) and differentiated (DM Edited) DM1 cells, as well as genomic DNA of the differentiated parental population (DM1 Not Edited), serving as control. Subsequently, libraries were prepared and sequencing was performed, achieving a mean coverage of ∼114,000×, enabling also the identification of rare events. The obtained reads were analysed using a software designed to identify single nucleotide variants (SNVs) and small insertions/deletions (indels), as well as their frequencies.^70^ Applying a 10X coverage filter, 20 potential off‐targets predicted for sgC3 and sg384 were investigated in detail (Table S2B). All identified sequence variations were also detected within the control population, indicating pre‐existing genomic variants rather than sequence changes caused by CRISPR/Cas9 treatment (Table S2C). The absence of off‐target effects in the analysed regions suggests the high specificity of the new sgC3/384 pair.

### 3.4 On‐target analysis

**FIGURE 2:**
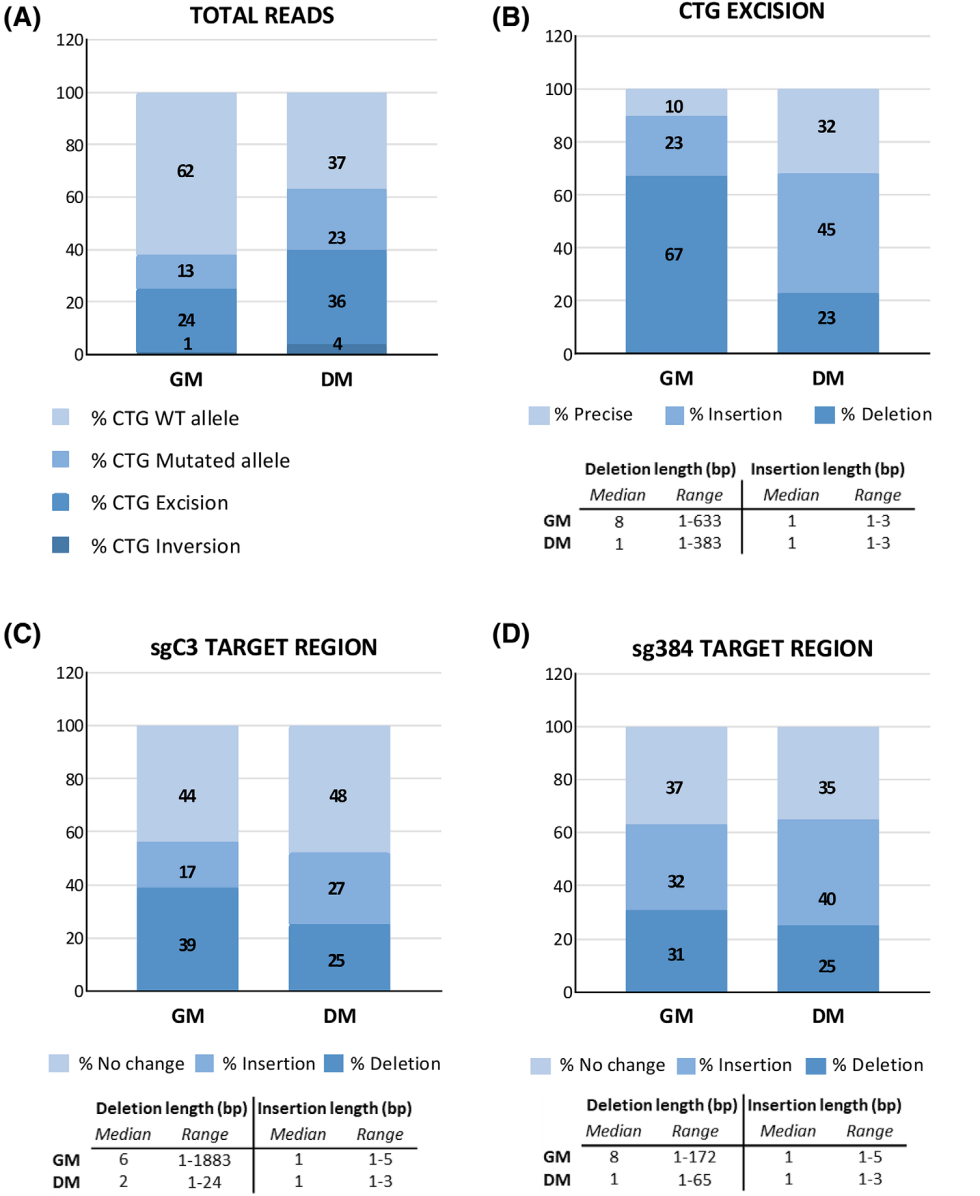
On‐target analysis of sgRNA C3/384‐edited myogenic cells derived from DM1 patients. Heterozygous DM1 cells transduced with lentiviruses expressing Cas9 and DOX inducible sgC3/384 were maintained in growth medium (GM) or induced to differentiate into myotubes in differentiation medium (DM) containing DOX for 5 days. Genomic DNA extracted from the cells was analysed by nanopore sequencing using Cas9‐mediated target‐enriched libraries (GM *n* = 2; DM *n* = 3). Editing accuracy and efficiency was estimated by alignment against the relevant reference sequence. Reads from each library for each cell state (GM or DM) were summed, and totals were normalised to represent 100%. The numbers within the columns indicate the percentage of reads identified for each category, relative to all reads aligning to the investigated region. (A) Total reads are expressed as percentage for each category. (B) Reads with CTG excision are expressed as percentage of reads without sequence changes (precise), insertions and deletions at the rejoining site, compared with the corresponding reference, relative to total CTG excision reads set at 100%. (C and D) Reads spanning sgRNA‐target sequences in the absence of CTG excision are expressed as percentage of reads showing no sequence variation (no change), insertions and deletions at the rejoining site, compared with reference, relative to total reads for each sgRNA region set at 100%. For investigating the precision of potential single cut events of either sgRNA, any sequence without CTG‐repeats excision was considered, pooling WT and mutated alleles. The tables below panels B, C and D indicate the middle value of the length distribution of all deletions or insertions (median) and the shortest and longest deletion or insertion lengths (range) measured in base pairs (bp), observed for each cell state.

To investigate editing accuracy and characterise sequence variations resulting from CRISPR/Cas9 activity guided by the new sgC3/384 pair, Cas9‐mediated target enrichment for Oxford Nanopore Technologies (ONT) was employed (Table S3A).^65^ This approach enabled the simultaneous analysis of on‐target cuts by both sgRNAs resulting in the excision of the CTG expansion, cut events induced by single sgRNAs, as well as non‐edited sequences within the same sample.^66^, ^67^ A total of five libraries from edited DM1 myogenic cell populations in both GM and DM were sequenced, yielding an overall sequencing coverage ≥ 140X for both cell states (Table S3B,C,D). Since libraries had different sequencing depth, we aggregated the samples for each cell state to provide a more representative analysis. The fraction of reads showing the intended CTG‐repeat excision indicated an editing efficiency of the sgC3/384 pair ranging from nearly 25% in GM to over 35% in DM cell populations (Figure 2A and Table S3C,D). Reads displaying inversions of the excised fragments were rarely found in both proliferating and differentiated cells (≤ 4%) (Figure 2A and Table S3C,D). A discrepancy was observed between the WT and mutated alleles in the number of reads without double‐cut editing activity. Notably, in GM, nearly five times more reads still containing CTG triplets originated from the WT allele compared with the mutant allele. In differentiated cells, this difference was much less pronounced (Figure 2A and Table S3C,D).

Among the reads with CTG excision, 10% in GM and 32% in DM showed precise cuts and perfect re‐joining after repeat removal (Figure 2B and Table S3C,D). The most common sequence changes at the rejoining site following triplet excision were deletions (67%) in GM and insertions (45%) in DM. Although most of the deletions observed in GM following CTG excision were shorter than 10 bp, three reads exhibited deletions overlapping with the *DMPK* coding sequence. In DM, deletions were less frequent and shorter, predominantly 1 basepair in length, with none overlapping the coding‐region (Figure 2B and Table S3C,D).

Analysis of sgRNA‐target sequences in the absence of CTG excision indicated an editing efficiency of approximately 50% and 65% for sgC3 and sg384, respectively, in both cell states (Figure 2C,D and Table S3C,D). It should be noted that editing activity from a single sgRNA followed by perfect re‐joining cannot be distinguished from an uncut target sequence. Notably, in both cell states, the predominant nucleotide among the insertions identified in the regions targeted by sgC3 and sg384 was identical to the fourth nucleotide upstream of the PAM sequence of the respective target site. Specifically, the most frequently occurring insertion in the sgC3 region was guanine, while in the sg384 region it was adenine. Deletions were more frequent (≥30%) and longer (median length ≥6 bp) in GM than in DM (incidence 25% and median length ≤2 bp) for both sgRNAs (Figure 2C,D and Table S3C,D). The analysis revealed one read with a deletion overlapping the protein‐coding sequence following the sgC3 cut in proliferating cells. The on‐target analysis suggested higher editing precision in the removal of CTG repeats by double cuts with sgC3/384 in differentiated myogenic cells compared with proliferating cells.

### 3.5 MyoAAV injection in homozygous DMSXL mice leads to attenuation of molecular defects in striated muscles

**FIGURE 3:**
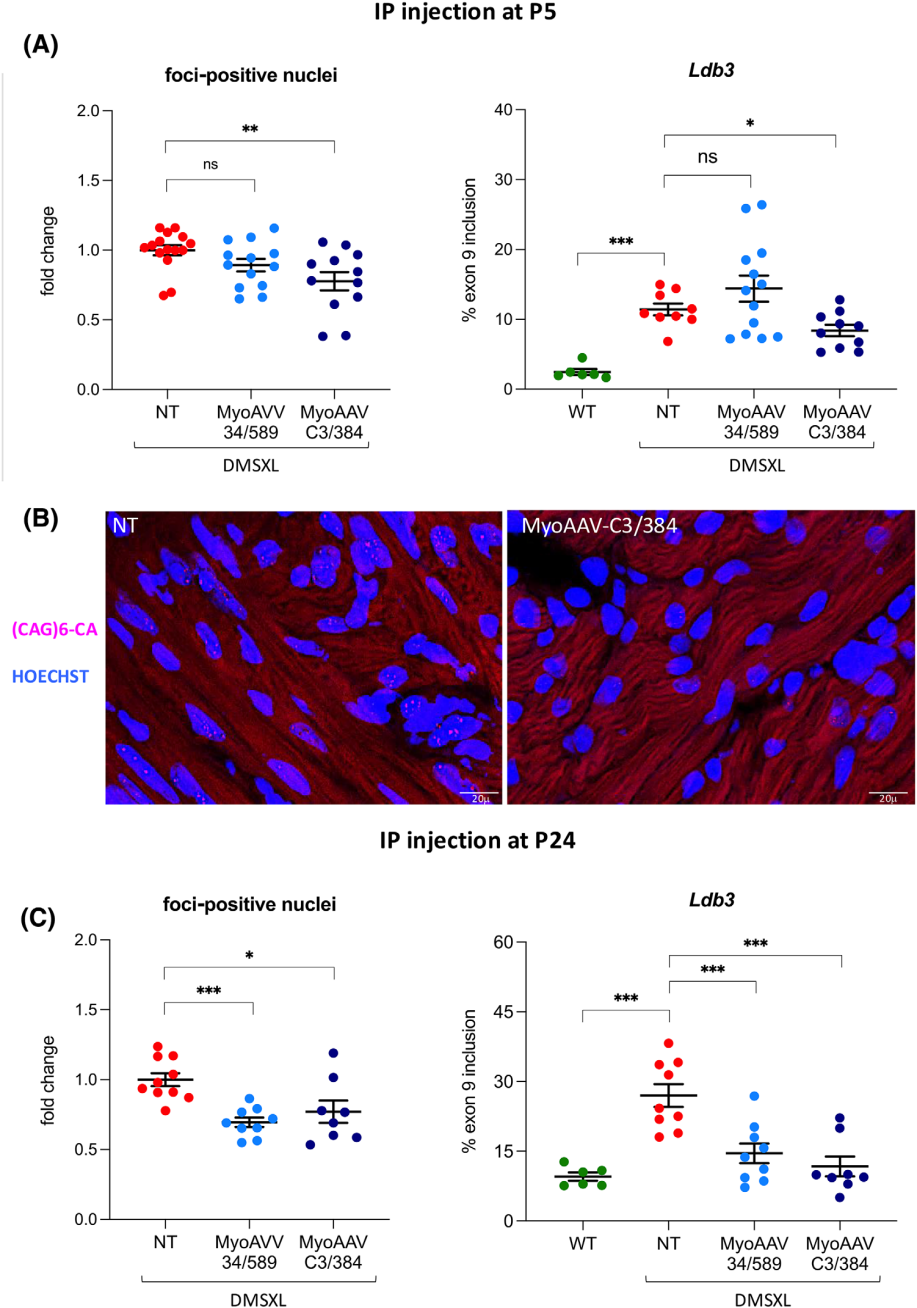
Improvement of the molecular phenotype in the heart of MyoAAV‐treated homozygous DMSXL mice. DMSXL mice were injected systemically with MyoAAVs carrying Cas9 and either sg34/589 or sgC3/384 pair at two different ages (P5 and P24). Four weeks later, the animals were sacrificed and heart sections were processed for RNA FISH using a (CAG)_6_‐CA probe to detect ribonuclear foci. In addition, AS was analysed by qRT‐PCR from RNA extracted from heart tissue. (A, left panel) The graph shows the decrease in foci‐containing nuclei in cardiac muscle of mice injected at P5, expressed as the average fold change in foci‐positive nuclei relative to untreated mice of the same age (NT), set as 1 (mean ± SEM). NT, *n* = 15; MyoAAV‐34/589, *n* = 13; MyoAAV–C3/384, *n* = 12. **p = .0048, ns = not significant. (A, right panel) AS of exon 9 of *Ldb3* transcript (NM_001039074.2) was analysed by qRT‐PCR using primers specific for each alternative isoform. The graph represents the mean percentage of exon inclusion in wild‐type (WT), untreated DMSXL homozygous control mice (NT) and mice injected at P5 (mean ± SEM). WT, *n* = 6; NT, *n* = 9; MyoAAV‐34/589, *n* = 1 3; MyoAAV–C3/384, *n* = 10. ***p = <.0001, *p = .0198. (B) Representative images of cardiac muscle sections from untreated (NT) or MyoAAV‐treated DMSXL mice subjected to RNA FISH analysis using a fluorescent (CAG)_6_CA probe; nuclei were counterstained with Hoechst dye. Bars: 20 µm. (C, left panel) The graph shows the decrease in foci‐containing nuclei in cardiac muscle of mice injected at P24, using the same expression and normalisation methods as described in A, left panel. NT, *n* = 10; MyoAAV‐34/589, *n* = 9; MyoAAV–C3/384, *n* = 8. ***p = .0001, *p = .0201, ns = not significant. (C, right panel) The graph represents the mean percentage of exon inclusion in wild‐type (WT), untreated DMSXL homozygous control mice (NT) and mice injected at P24, using the same expression and normalisation method as described in A, right panel. WT, *n* = 6; NT, *n* = 9; MyoAAV‐34/589, *n* = 9; MyoAAV–C3/384, *n* = 8. ***p < .001. The statistical analyses presented in the figure were conducted using an unpaired *t*‐test, with Welch's correction applied where appropriate. Each dot represents a single individual.

For in vivo expression of the CRISPR/Cas9 machinery, we took advantage of a recently identified AAV capsid protein variant (MyoAAV 2A) that confers high tropism to striated muscle.^57^ Editing efficiency and muscle tropism of MyoAAV–U6–34/589 and MyoAAV–CK8–Cas9 compared with the previously used AAV9–U6‐34/589 and AAV9–CK8‐Cas9 vectors was first tested in DMSXL hemizygous mice by systemic injection at P5. Four weeks after injection, mice were sacrificed and striated muscles (heart, diaphragm, TA and gastrocnemious) and liver were analysed. All striated muscles analysed showed higher editing (Figure S5A) and expression levels of the CRISPR/Cas9 components (Figure S5B) in MyoAAV injected mice compared with AAV9 injected mice, while this was not observed in the liver. This is indeed expected, because MyoAAVs have also been selected for liver de‐targeting to avoid liver toxicity.^57^ All the following experiments were performed with MyoAAVs. Homozygous DMSXL mice were injected with either MyoAAV–U6‐34/589 or MyoAAV–U6‐C3/384 both in combination with MyoAAV–CK8–Cas9 at P5 or P24 (Figure S6). The two different ages were selected with the aim of verifying the efficiency of the treatment in neonates and in young adult mice. While treating neonates has the advantage to intervene at an early stage, it also may increase the risk of mortality in an already critical window (pre‐weaning) for the survival of DMSXL homozygous mice. Thus, post‐weaning treatment could be a safer choice. A survival analysis of animals injected at P5 up to 17 weeks after injection indicated that the difference in mortality rate between CRISPR/Cas9‐ or PBS‐injected mice was not statistically significant (Figure S7). All the mice injected at P24 survived until sacrifice. Four weeks after injection, mice were sacrificed and the heart and TA muscles analysed to determine the number of nuclear foci in tissue sections and to quantify the AS of the *Ldb3* transcript, previously identified as one of the most altered in homozygous DMSXL muscles.^32^ In the heart, we found a significant improvement of both foci and AS in most mice injected at both P5 and P24 (Figure 3A–C). The observed decrease of foci‐positive nuclei ranges from 10 to 30% depending on the age of injection and the sgRNA pair used. This reduction is lower compared with that observed in DM1 myogenic cells, yet in some cases is sufficient to obtain improvement of *Ldb3* transcript AS. Dot‐blot analysis of CUG‐repeated transcripts from the heart tissues of mice injected with MyoAAV‐34/589 at P24 revealed more than 30% decrease in transcript accumulation (Figure S8A,B). However, similarly to edited DM1 cells, total *DMPK* mRNA levels remained unchanged following CRISPR/Cas9 treatment (Figure S8C). In mice injected with MyoAAV‐34/589, *SIX5* mRNA expression was also unaffected by the treatment (Figure S4B). We further quantified the percentage of CTG excision in the heart tissues of the same mice by absolute qPCR of genomic DNA using the standard curve method. We observed an average deletion efficiency of 3% in mice injected at P24 (Figure S8D), a value lower than that measured in DM1 cells. Due to the low editing efficiency, we could not apply the ONT sequencing of genomic DNA from mouse tissues. It should be considered, however, that the results obtained by qPCR are not comparable to the on‐target sequencing results performed in DM1 cells, since deletions including primer recognition sequences would not be amplified by qPCR and editing efficiency could be underestimated.

**FIGURE 4:**
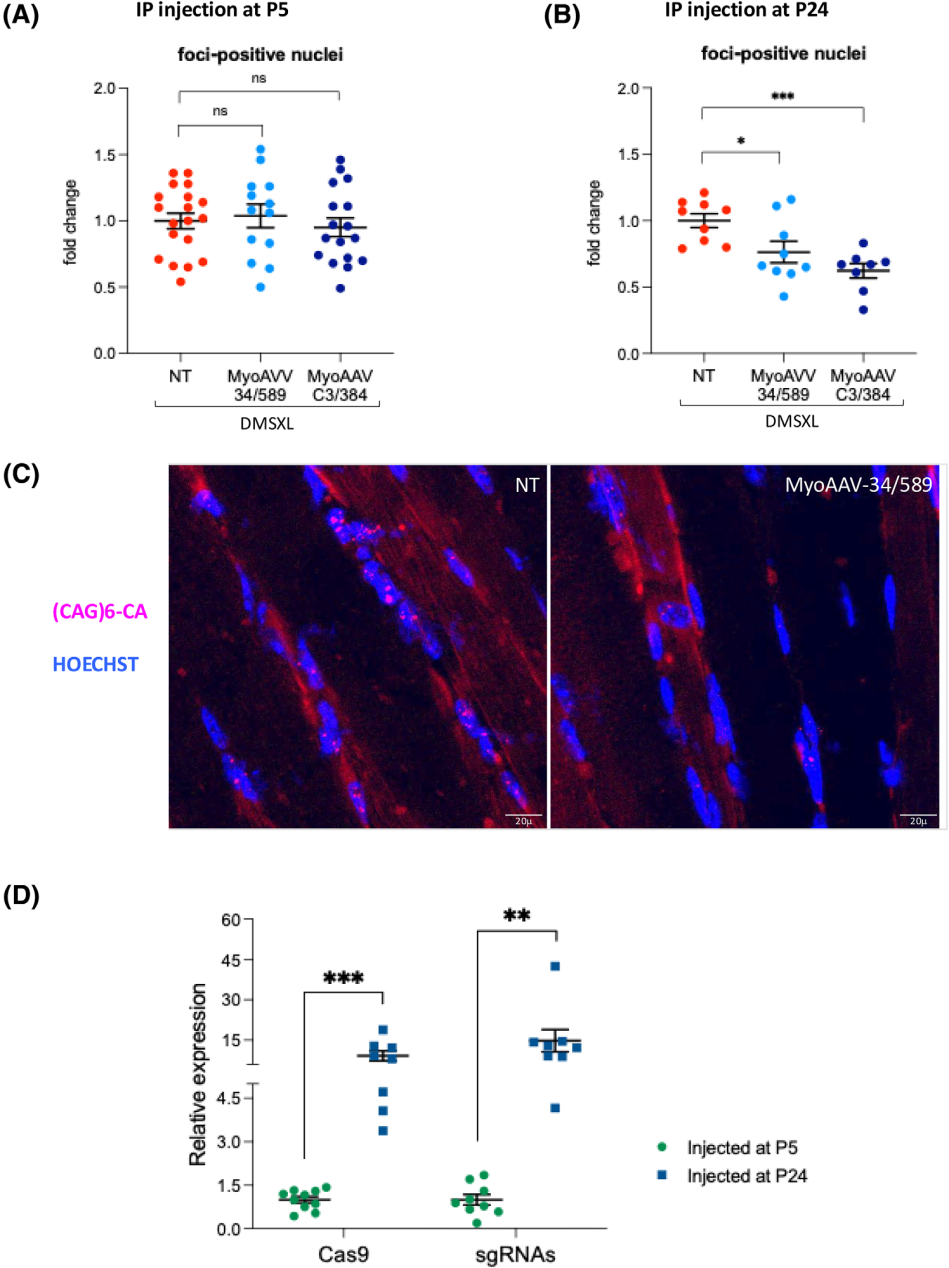
Reduction of ribonuclear foci in the TA muscle of MyoAAV‐treated homozygous DMSXL mice. TA sections of DMSXL mice treated as in Figure 3 were analysed by RNA FISH to detect the number of foci‐positive nuclei and by qRT‐PCR to measure Cas9 and sgRNA expression. (A) The graph represents the decrease of the foci‐containing nuclei in TA muscle of MyoAAV‐treated mice injected a P5, expressed as the average fold change in foci‐positive nuclei relative to untreated mice of the same age (NT), set as 1 (mean ± SEM). NT, *n* = 19; MyoAAV‐34/589, *n* = 13; MyoAAV–C3/384, *n* = 17; ns = not significant. (B) The graph shows the decrease in foci‐containing nuclei in TA muscle of mice injected at P24, using the same expression and normalisation methods as described in A. NT, *n* = 9; MyoAAV‐34/589, *n* = 9; MyoAAV–C3/384, *n* = 8. *p = .0265, ***p = .0002. (C) Representative images of TA muscle sections from untreated (NT) or MyoAAV‐treated DMSXL mice subjected to RNA FISH analysis using a fluorescent (CAG)_6_CA probe; nuclei were counterstained with Hoechst dye. Bars: 20 µm. (D) qRT‐PCR analysis of Cas9 mRNA and sgC3/384 in TA muscles of injected mice, normalised to *Rer1* mRNA, and shown as expression in individuals injected at P24 relative to those injected at P5, set as 1 (mean ± SEM). The primer pair used for sgRNA analysis can amplify both sgRNAs. P5, *n* = 12; P24, *n* = 8. ***p < .00021, **p < .0030. The statistical analyses presented in the figure were conducted using an unpaired t‐test, with Welch's correction applied where appropriate. Each dot represents a single individual.

In TA muscles no significant decrease of nuclear foci was observed in mice injected at P5, while the number of foci decreased in mice injected at P24, and the change was particularly remarkable when using the sgC3/384 pair (Figure 4A–C). In addition, no improvement of *Ldb3* AS alteration was observed in TA muscles at both ages of treatment. When the expression levels of the CRISPR/Cas9 components were analysed in the TA muscles of mice injected with MyoAAVs carrying the sgC3/384 pair, a strong difference between the injection times was observed, since both Cas9 and sgRNAs were expressed at significantly higher levels in mice treated at P24 than in mice treated at P5 (Figure 4D). Moreover, Cas9 and sgRNAs were found to be expressed at higher levels in the hearts relative to TA muscles (Figure S9). These data may help to explain the age‐ and organ‐dependence of the observed nuclear foci and AS recovery.

### 3.6 MyoAAV injection in homozygous DMSXL mice leads to improvement of body weight and muscle strength

**FIGURE 5:**
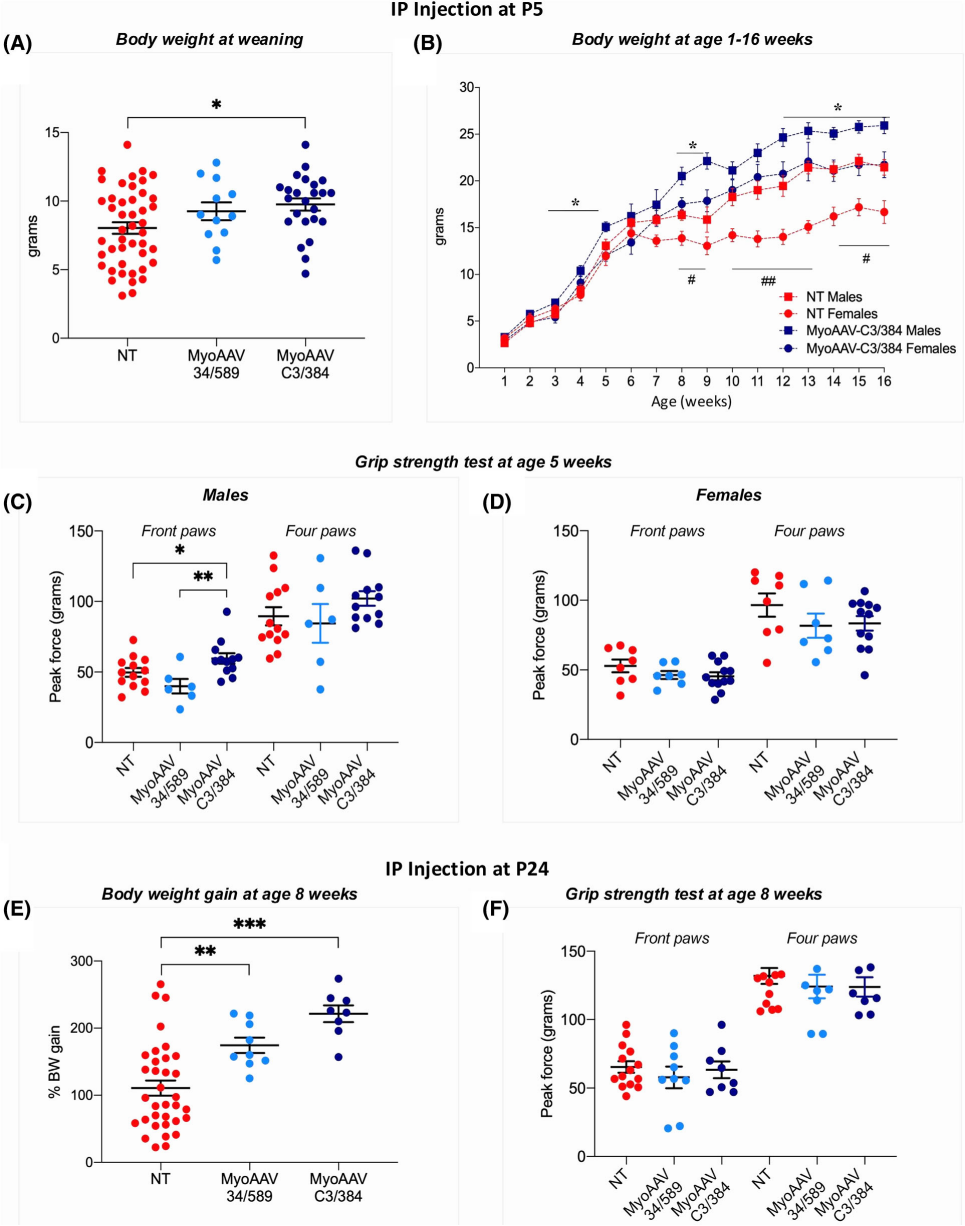
Increase of body weight and grip strength in MyoAAV‐treated homozygous DMSXL mice. DMSXL homozygous mice were injected systemically at P5 (A–D) or P24 (E and F) with MyoAAV carrying Cas9 and either sg34/589 or sgC3/384 pair. (A) Body weight measured at weaning (4 weeks of age), about 4 weeks after treatment, differed between NT (*n* = 45), MyoAAV‐34/589 (*n* = 12) and MyoAAV–C3/384 (*n* = 24) groups, one‐way ANOVA treatment factor *F*_(2,78) _= 3.74, *p* = .028; *p < .05 (Tukey's post‐hoc test). Mean ± SEM and individual data points of male and female mice. (B) Body weight measured from 1 to 16 weeks of age in males and females of NT and MyoAAV–C3/384 groups (for individual's body weight curves and related statistical analyses, see Figure S10). Not all mice were weighed at each time point (for n's at each age see Table S4). Slopes from simple linear regression analysis are significantly different (*p* < .0001) between NT and MyoAAV–C3/384 groups in both males (*F*_(1,383) _= 18.79) and females (*F*_(1,216) _= 47.11); *p < .05, NT vs. MyoAAV–C3/384 in males, and #*p* < .05, ##*p* < .005 in females (multiple *t*‐tests). (C and D) Grip strength measured as peak force in grams at 5 weeks of age in front and four paws of males of NT (*n* = 13), MyoAAV‐34/589 (*n* = 6) and MyoAAV–C3/384 (*n* = 12) groups (C) and females of NT (*n* = 8), MyoAAV‐34/589 (*n* = 7) and MyoAAV–C3/384 (*n* = 12) groups (D). (C) Front paws: one‐way ANOVA treatment factor *F*_(2,28) _= 5.47, *p* = .009; *p = .05, **p < .005 (Fisher's PLSD post‐hoc test). Mean ± SEM and individual data points. (E) Body weight gain at 8 weeks of age (4 weeks after treatment), expressed as percentage increase from injection time, differed between NT (*n* = 34), MyoAAV‐34/589 (*n* = 9) and MyoAAV–C3/384 (*n* = 8) groups; one‐way ANOVA treatment factor *F*_(2,48) _= 14.08, *p* < .0001; **p < .005, ***p < .0001 (Fisher's PLSD post‐hoc test). Mean ± SEM and individual data points of male and female mice. (F) Grip strength measured as peak force in grams at 8 weeks of age in front and four paws of NT (*n* = 14), MyoAAV‐34/589 (*n* = 9) and MyoAAV–C3/384 (*n* = 8) groups of male and female mice. Data sets not labelled by asterisk symbol are ‘not statistically significant’.

Among the many phenotypical alterations, DMSXL homoxygous mice exhibit strongly reduced size and body weight and muscle weakness.^32^ To evaluate the effects of MyoAAV treatment in vivo, body weight and grip strength were measured in mice injected at P5 or P24, approximately 4 weeks post‐injection (Figure S6). At weaning (age 4 weeks), body weight of DMSXL homozygous mice injected at P5 was increased of 15% in mice treated with MyoAAV‐34/589 sgRNA pair and of 21% when injected with MyoAAV–C3/384 sgRNA pair compared with NT animals (Figure 5A). Importantly, the effect on body weight improvement of MyoAAV carrying the sgRNA C3/384 pair injected at P5 persisted over time up to at least the age of 16 weeks in both sexes (Figures 5B, S10 and Table S4). A significant increase in grip strength upon MyoAAV–C3/384 sgRNA pair injection (+20%) compared with NT and MyoAAV‐34/589 sgRNA pair groups was observed, at age 5 weeks, only in front paws of male DMSXL homozygous mice treated at P5. However, differences were not statistically significant when the grip strength was measured with four paws or in females (Figure 5C,D).

A clear effect of treatment was observed, after 4 weeks, in body weight gain of DMSXL homozygous mice injected at P24. In fact, at 8 weeks of age, mice injected with both sgRNA pairs showed a significant increase in body weight gain compared with NT group, with the sgRNA C3/384 pair showing a greater increase compared with the sgRNA 34/589 pair (Figure 5E). The MyoAAV injection at P24 did not seem to improve grip strength at age 8 weeks (Figure 5F). Overall, data obtained in vivo indicate that the newly characterised sgRNA C3/384 pair shows a more favourable rescue profile compared with the previously employed sgRNA 34/589 pair.^36^ Moreover, it appears that an early treatment could lead to a long‐lasting increase of body weight.

### 3.7 MyoAAV injection in homozygous DMSXL mice leads to a rescue of body composition phenotype

**FIGURE 6:**
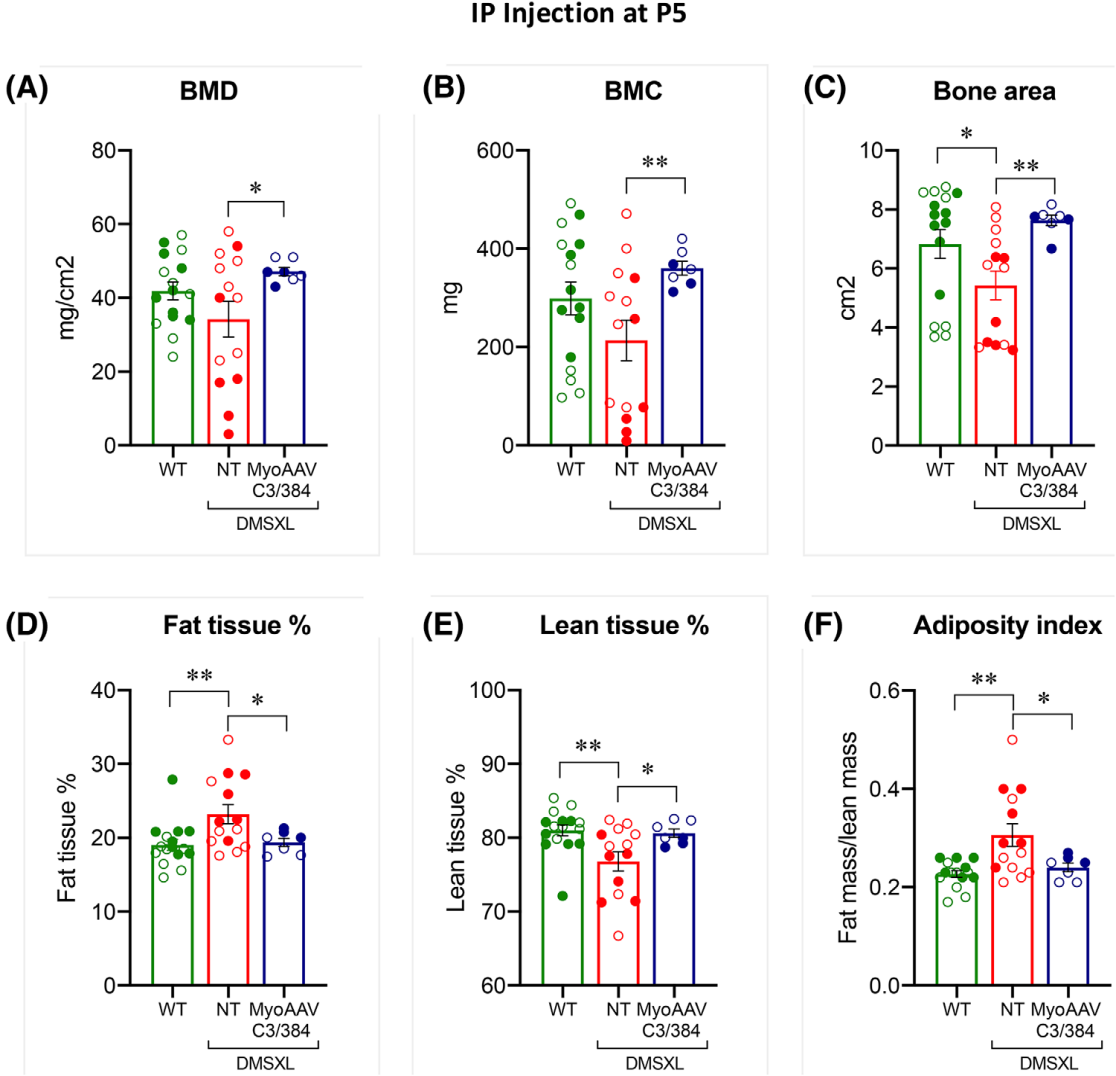
Improvement of bone health and body composition parameters in MyoAAV–C3/384‐treated homozygous DMSXL mice. A group of DMSXL male and female homozygous mice at age P5 received a systemic injection of MyoAAV vectors carrying Cas9 and sgC3/384 pair (*n* = 7), and underwent whole‐body DEXA analysis at 16 weeks of age. Control animals were wild‐types (WT, *n* = 16) and untreated DMSXL homozygous mice (NT, *n* = 14) of both sexes. All data are represented as mean ± SEM and individual data points; data from male (open symbols) and female (filled symbols) mice are pooled. (A–C) Bone health parameters: bone mineral density (BMD) (A), bone mineral content (BMC) (B) and bone area (C). (A) BMD: MyoAAV–C3/384 vs. NT, unpaired *t*‐test with Welch's correction, *t* = 2.595, df = 14, *p = .02. (B) BMC: MyoAAV–C3/384 vs. NT, unpaired *t*‐test with Welch's correction, *t* = 3.371, df = 15, **p = .004. (C) Bone area, WT vs. NT, Mann–Whitney test, *U* = 53, *p = .013; MyoAAV–C3/384 vs. NT, unpaired *t*‐test with Welch's correction, *t* = 4.25, df = 16, ***p = .0006. (D–F) Body composition parameters: fat tissue percentage (D), lean tissue percentage (E) and fat/lean mass ratio (adiposity index, F). (D) Fat tissue %: WT vs. NT, Mann–Whitney test, *U* = 46, **p = .0052; MyoAAV–C3/384 vs. NT, unpaired *t*‐test with Welch's correction, *t* = 2.684, df = 17.14, *p = .015. (E) Lean tissue %: WT vs. NT, Mann–Whitney test, *U* = 45, **p = .0045; MyoAAV–C3/384 vs. NT, unpaired *t*‐test with Welch's correction, *t* = 2.684, df = 17.14, *p = .015. (F) Adiposity index, WT vs. NT, unpaired *t*‐test with Welch's correction, *t* = 3.265, df = 15.37, **p = .0051; MyoAAV–C3/384 vs. NT, unpaired *t*‐test with Welch's correction, *t* = 2.643, df = 16.41, *p = .017.

Alterations in body composition have been described in DM1 patients.^5^ DMSXL homozygous mice subjected to DEXA showed abnormalities in body composition, a novel phenotype uncovered in this strain (Golini et al. manuscript in preparation). These alterations were rescued after treatment at P5 with MyoAAV–C3/384. In particular, a significant increase in BMD and BMC, as well as bone area were observed at 16 weeks of age in treated DMSXL homozygous animals compared with NT, reaching values similar to WT (Figure 6A–C).

Additionally, DMSXL homozygous NT mice showed, compared with WT, a significant increase of fat tissue percentage and a corresponding decrease of lean tissue percentage, as expected (Figure 6D,E), resulting in a shift in body composition towards a greater amount of fat at the expense of lean tissue. This was further evidenced by the increased fat/lean mass ratio in NT animals (adiposity index; Figure 6F). All the parameters recovered to WT levels in DMSXL homozygous mice injected at P5 with MyoAAV–C3/384 (Figure 6D–F).

### 3.8 Decrease of nuclear foci by gene editing‐dependent and ‐independent mechanisms

**FIGURE 7:**
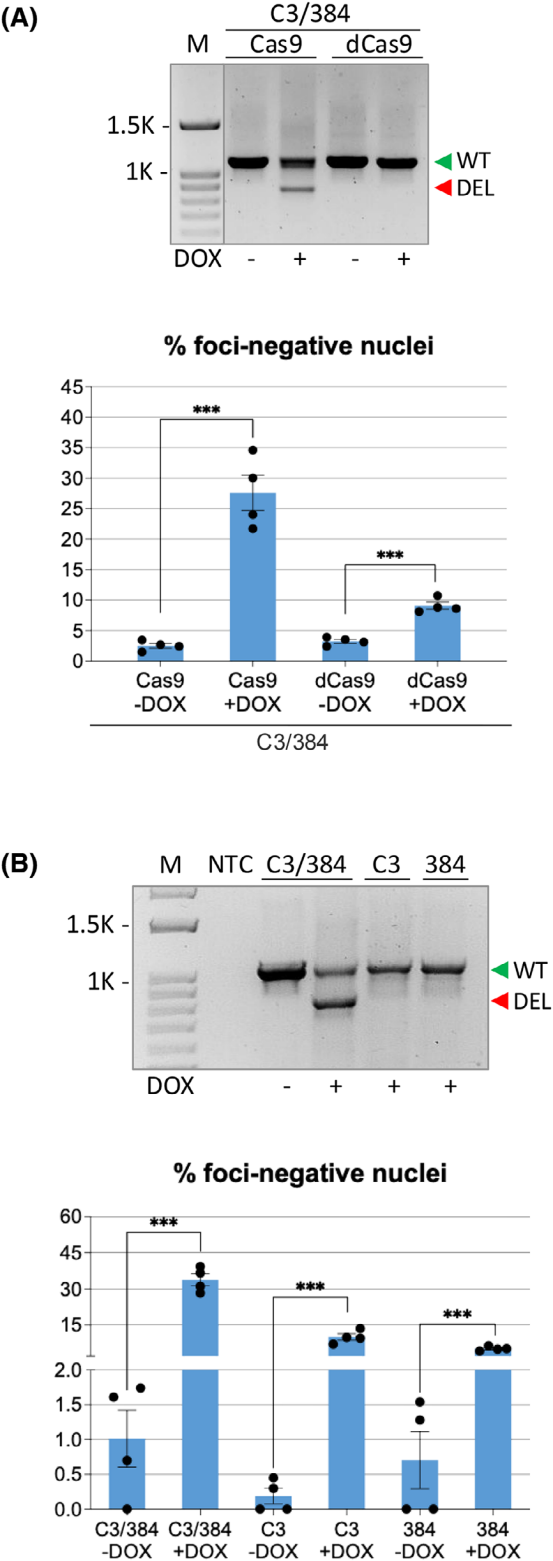
Role of nuclease‐defective Cas9 and single sgRNA–Cas9 complexes on reduction of ribonuclear foci. DM1 cells transduced with lentiviruses expressing Cas9 or dCas9 and DOX‐inducible sgC3/384 pair (A) or Cas9 and DOX‐inducible single sgC3 or sg384 (B) were untreated or treated with DOX in GM for 5 days and analysed by PCR (top panels) and FISH assay (bottom panels). For PCR analysis, genomic DNA was extracted and analysed as described in Figure 1A. The green triangle indicates undeleted wild‐type (WT) allele‐derived amplicons, the red triangle indicates the expected CTG‐deleted products (DEL) from both wild‐type and mutated alleles. For FISH analysis, histograms show the mean percentage of nuclei containing no foci (mean ± SEM). The statistical analyses presented in the figure were conducted using an unpaired *t*‐test, with Welch's correction applied where appropriate. *n* = 4; ***p < .001. Each dot represents an individual sample from independent experiments.

Based on the findings presented in the previous sections, a discrepancy arises. The efficiency of the on‐target excision events in DM1 cells, as estimated from the processing of both the WT and the mutated alleles, is lower than expected, when compared with the remarkable reduction of nuclear foci and the decreased accumulation of the CUG‐repeated *DMPK* transcript. This is even more evident in the hearts of treated DMSXL mice where we measured an average 3.2% of CTG deletion. However, in spite of the modest detectable editing, we observed a significant recovery of body weight, muscle strength and body composition in DMSXL treated mice. Therefore, we hypothesised that CRISPR/Cas9‐mediated mechanisms other than genomic CTG deletion could contribute to the reduction of *DMPK* transcript and the decrease in nuclear foci, observed both in vitro and in vivo. First, we tested the effect of a dCas9, devoid of nuclease activity, by transfecting Cas9‐ and dCas9–sgC3/384 ribonuclear complexes in DM1 cells and analysed the number of foci. We found that dCas9 ribonuclear complexes were able to reduce the number of foci and led to an increase of cells with foci‐free nuclei, even in the absence of the CTG‐repeat deletion, albeit at lower efficiency compared with the complexes containing editing‐proficient Cas9 (ninefold vs. 28‐fold over the cells transfected with control HPRT sgRNA) (Figure S11A, left and central panel). Notably, dCas9–ribonuclear complexes also induced a decrease in the fraction of nuclei containing more than 5 foci (twofold decrease with dCas9 vs. threefold decrease with Cas9 compared with the cells transfected with control HPRT sgRNA) (Figure S11A, right panel). The effect of dCas9 observed in transient transfection was confirmed in transduced DM1 cells. Cells infected with lentiviruses expressing Cas9 or dCas9 along with lentiviruses expressing DOX‐inducible sgC3/384 were treated with DOX in GM for 5 days and analysed by FISH analysis. The results indicate that DM1 cells stably expressing dCas9 are also characterised by a significant increase in the number of foci‐free nuclei in the absence of CTG‐repeat deletion, although at lower levels compared with DM1 cells expressing nuclease‐proficient Cas9 (approximately threefold for dCas9 and 11‐fold for Cas9, between DOX treated and untreated cells) (Figure 7A). Moreover, the fraction of nuclei containing more than 5 foci was reduced approximately twofold with both Cas9 and dCas9 (Figure S12A). To exclude that this could reflect a differential expression of Cas9s and sgRNAs in the two DM1 cell populations, we evaluated the abundance of the CRISPR/Cas9 components in DOX‐induced cells and found no significant difference in transcript accumulation of the two Cas9s and sgRNAs (Figure S12C,E). Dot‐blot analysis of the mutated *DMPK* transcript in dCas9‐expressing cells did not reveal a significant decrease in mutated *DMPK* transcript accumulation, unlike what was observed in cells expressing Cas9 (Figure S3B,D), suggesting that a functional nuclease activity is important to achieve a strong inhibitory effect in DM1 cells. The accumulation of total *DMPK* transcript also remained unaltered in cells treated with dCas9‐sgC3/384 (Figure S3F).

To also evaluate sgRNA‐mediated events independent from CTG‐repeat deletion, DM1 cells were co‐transfected with a single sgRNA along with Cas9 protein, or transduced with lentiviruses expressing Cas9 and a single sgRNA. In transient transfection assays (Figure S11B), as well as in transduced DM1 cells (Figure 7B), the expression of Cas9 and of a single sgRNA led to an increase of cells with foci‐free nuclei, particularly when using the sgRNA binding upstream of the CTG repeats (∼fivefold increase in sgC3 transiently transfected cells and eightfold increase in sgC3‐transduced cells). In transduced cells, expressing similar levels of Cas9 and inducible sgRNAs (Figure S12D,F), we also observed a significant decrease in the fraction of nuclei containing more than 5 foci (Figure S12B). However, an effect of single sgRNA editing on mutated and total *DMPK* transcript accumulation could not be detected (Figure S3C,E,G).

## 4 DISCUSSION

Following our previous studies focused on the design and application of the CRISPR/Cas9 strategy to remove the pathogenetic CTG‐triplet amplification in DM1 patient‐derived cells,^28^, ^36^ here we describe a further progress towards the application of this approach in vivo in DMSXL mice, an established model of DM1. To bypass the strong limitation of the low editing efficiency in vivo, we identified novel tools that allowed to improve the expression levels and the activity of the CRISPR/Cas9 complexes: more efficient sgRNA guides were designed, scAAVs were used to increase sgRNA expression, and a myotropic AAV capsid protein variant was employed to direct CRISPR/Cas9 components specifically to striated muscles. By applying these combined tools, we achieved an improvement of molecular defects in striated muscles. In the heart, we found a significant decrease of foci and splicing in most mice injected at both P5 and P24. In TA muscles we detected no significant decrease of nuclear foci in mice injected at P5, while foci number decreased significantly in mice injected at P24, possibly due to the higher expression levels of CRISPR/Cas9 components in young adult mice. The rescue of molecular defect was accompanied by improvement of phenotypical alterations following systemic treatment. Body weight at weaning of mice injected at P5 was increased compared with untreated animals, reaching statistical significance and persistence over time in both sexes for the sgRNA C3/384 pair. We also observed a significant increase in grip strength, although only in front paws of male mice injected at P5 with the sgRNA C3/384 pair. Notably, the sgRNA C3/384 pair appears to be more effective in many aspects compared with the sgRNA 34/589 pair. This efficiency could be associated to the higher editing activity measured by on‐target sequencing analysis, but a direct comparison should be demonstrated.

Interestingly, we showed an improvement of abnormal body composition parameters. The rescue of this newly described phenotype of DMSXL homozygous mice could be also relevant for DM1 patients presenting alterations of these parameters.^79^ Indeed, increased fat mass associated with muscle weakness has been repeatedly reported in DM1, and parameters measured by DEXA might be used as reliable biomarkers to assess efficacy of therapies.^5^ The muscle targeted treatment used in this study, may also reflect on fat, bone health and metabolism. Since muscle is the most abundant tissue in the body, it is possible that even a small improvement in its physiology is beneficial for bones and other body compartments, and ultimately for metabolism.^80^, ^81^, ^82^ Although editing efficiency needs to be further improved, our results show that the CRISPR/Cas9 gene therapy strategy can be applied successfully in this DM1 mouse model. Moreover, the exploration of a gene therapy approach targeting tissues/organs other than muscles (e.g. nervous and metabolism‐related systems), should be implemented given the multi‐systemic nature of DM1.

In treated DM1 cells we observed that expression level of the mutated *DMPK* transcript and, correspondingly, the ribonuclear foci, decreased remarkably despite a relatively low CTG‐deletion efficiency. This was much more evident in homozygous DMSXL hearts, where only mutated transcripts are expressed and CTG‐deletion efficiency is much lower compared with DM1 cells. These data suggest that other CRISPR/Cas9‐dependent mechanisms may be involved in mutated *DMPK* transcript down‐regulation. A specific reduction of the *DMPK* mutated transcript in Cas9‐treated cells could result from CTG contraction at genomic level. Indeed it was described that either DSB or single strand break induced by Cas9, TALEN, ZNF and Cas9‐nickase can lead to CTG contraction through recruitment of different repair mechanisms.^51^, ^83^, ^84^, ^85^ Since Cas9/dCas9–sgRNA complexes have been shown to bind RNA,^86^, ^87^, ^88^ post‐transcriptional mechanisms may help explain the observed reduction in foci. For instance, having Cas9 been implicated in cleavage of ssRNA,^44^ although at low efficiency, it is possible that the binding of Cas9 complexes to *DMPK* transcripts induce their destabilisation. Furthermore, the binding of CRISPR/Cas9 complexes to *DMPK* mRNA may hinder MBNL protein interaction with the mutated transcript, thereby interfering with foci formation. This effect could also account for the reduction in foci observed in experiments with single sgRNAs or dCas9, where no detectable decrease in transcript accumulation was observed. All these mechanisms, not yet fully elucidated, could potentially contribute to the remarkable improvement of DM1‐associated molecular and phenotypic alterations despite a low efficiency of the anticipated CTG deletion in both DM1 cells and mouse tissues.

Although total *DMPK* transcript levels remained unchanged, we found that editing CTG expansions in DM1 cells leads to a restoration of *SIX5* mRNA accumulation, which is reduced in the analysed DM1 cells compared with normal controls. Decreased accumulation of *SIX5* transcript was previously described in patient‐derived DM1 cells and in skeletal muscle and myocardium from DM1 patients,^23^, ^24^ likely due to CTG expansion‐induced modifications of nearby chromatin.^18^, ^19^, ^20^, ^21^ However, some studies report no changes in *SIX5* mRNA expression levels in DM1 embryonic stem cells and patient‐derived myoblasts compared with controls, nor in gene‐edited DM1 cells.^51^, ^89^ These discrepancies could arise due to the use of different cell types and/or targeting of different regions for deletion.

For the clinical translation of CRISPR/Cas9 technologies, a thorough assessment of specificity, safety and efficacy in gene editing is imperative. The gene editing of sgC3 and sg384 in DM1‐myogenic cells was analysed through ultra‐deep sequencing, revealing no evidence of off‐target activity in the predicted off‐target genomic sites, neither in GM nor in DM. Although an unbiased, genome‐wide assessment of specificity would be ideal, techniques such as whole genome sequencing have inherent limitations, including low signal‐to‐noise ratio, limited sensitivity for rare variants and high costs due to the need for extensive coverage (20–60×).^90^ Moreover, off‐target mutations occur at low frequencies, making it challenging to distinguish them from the background rate of de novo mutations.^91^

On‐target analysis using third‐generation sequencing indicates enhanced editing efficiency and accuracy of the newly designed sgC3/384 pair during CTG‐repeat removal. Both the fraction of double‐cut events and the number of perfectly cut‐and‐repaired reads increased compared with the results previously obtained for sg34/589 using amplicon sequencing and droplet digital PCR.^36^ However, it is important to consider the differences between ONT and amplicon sequencing‐based approaches when making this comparison. Inversions caused by sgC3/384 double cuts were found, consistent with our prior qPCR‐based results for the sg34/589 pair.^36^ The generation of expanded CAG repeats can lead to the translation of potentially toxic proteins, via repeat‐associated non‐AUG (RAN) translation.^16^, ^92^ However, the observed recovery of phenotype, in vitro as well as in vivo, suggests that the overall positive effects outweigh the potential negative consequences. While low coverage is a recognised drawback of the ONT platform, its advantage lies in its independence from read length or sequence, including low‐complexity repeat regions.^93^, ^94^ Therefore, all expected events, both edited and non‐edited, as well as unwanted events, such as long deletions, could be analysed within the same sample. Our ONT analysis has limitations. First, due to the size disparities between WT and mutated alleles, we cannot rule out that the observed differences in CTG excision between WT and mutated alleles may, to some extent, be attributed to technical difficulties in the sequencing of very long CTG repeats, rather than differences in editing efficiency. That could lead to a slight overestimation of editing activity. However, even assuming a technical bias, the disparity in the number of retained CTG‐reads in the WT allele was multiple times higher in GM than in DM culture conditions. Since the repeat lengths of the WT and mutated alleles can be assumed very similar across the two conditions, the discrepancy was likely genuine, at least in proliferating cells. Second, the obtained ONT libraries resulted in different sequencing depths. To provide a more representative analysis, we chose to aggregate the libraries for each cell state and, consequently, conclusions are based on pooled datasets. Third, due to operative constraints, ONT sequencing of the unedited cell population was not performed. Consequently, sequence variants, such as internal deletions or expansions of the CTG repeats, that existed prior to editing activity may have eluded our analysis. Although deletions shorter than 10 bp were common at sgRNA target sites, proliferating edited cells exhibited deletions ranging from several hundred to nearly 2000 bp in length. Such substantial sequence alterations would have gone unnoticed in a deep sequencing amplicon setup due to technical restrictions. While analysis at sgRNA cut sites suggested an editing efficiency of approximately 50% for sgC3 and sg384, evaluation of ribonuclear foci reduction due to single‐sgRNA cut events in GM showed a decrease of less than 20%. It is reasonable to assume that editing due to a single DSB is less efficient in reducing foci than a double cut. Studies have demonstrated that numerous target sites show repeated insertions of the same bases, implying a non‐random selection of the inserted nucleotide.^95^ Consistent with the previous findings,^36^, ^96^ in sgC3/384 edited myogenic cells, we found a high frequency of insertion of a nucleotide identical to that located at position −4 from the PAM sequence, typically the nucleotide upstream of the cleavage site. Due to the low editing efficiency in vivo, the ONT genomic analysis described for DM1 myogenic cells could not be performed in mouse heart tissue. However, the assessment of the occurrence of unintended events is more relevant in a human cell context in view of the clinical application of this gene therapy strategy.

DSBs elicited by Cas9 nucleases are mainly repaired by non‐homologous end joining (NHEJ), active in all cell‐cycle phases, including post‐mitotic states predominant in DM1‐affected tissues like striated muscles and the central nervous system. The processing of free DNA ends before religation can result in short deletions or insertions,^97^, ^98^ but this is not relevant in our context as the CTG‐repeat region is in the UTR of the *DMPK* gene, and *DMPK* transcripts and proteins are normally produced in edited cells.^28^ Although nuclease‐induced DSBs at on‐target sites may sometimes lead to large deletions and translocations thus enhancing genotoxic risk,^55^, ^99^, ^100^ we observed these events mainly in proliferating cells, in line with our previous sg34/589 on‐target analysis.^36^ The local chromatin environment influences the DSB repair pathway choice: NHEJ prefers euchromatin, while microhomology‐mediated end joining (MMEJ) is higher in heterochromatin, marked by H3K9me2, lamina‐associated domains and late replication.^101^ Differences in chromatin structure between WT and mutated *DMPK* alleles, with expanded CTG‐repeats inducing CpG methylation and heterochromatinisation,^12^, ^20^, ^102^, ^103^ may contribute to the variation in editing efficiency, particularly during the S/G2 phases of actively dividing cells, where MMEJ is predominantly active.^98^, ^104^

The in vivo CRISPR/Cas9 activity described here is directed mostly to non‐dividing myocytes/myofibers of the striated muscles in treated mice. It is unlikely that gene editing occurs in satellite cells since the CK8 promoter is transcriptionally inactive in dividing cells and in non‐muscle tissues in vivo, while it is functional in all skeletal muscles, as well as in cardiac muscle.^105^, ^106^ Concerning the occurrence of possible toxic effects of gene editing, we observed no significant difference in mortality rate between CRISPR/Cas9 injected and control mice up to 4 months of age. Rather, the treated mice showed improvements in body weight, muscle strength and body composition. Therefore, an editing‐dependent genotoxic effect is unlikely to manifest in this case.

## 5 CONCLUSIONS

The CRISPR/Cas9 technology is a powerful tool for gene therapy application. However, adverse genomic events have been described^100^ and need to be carefully evaluated. While the off‐target events can be predicted and easily detectable, undesired on‐target events are more difficult to be determined and likely underestimated. Although complex and time consuming, a combination of different approaches is desirable to highlight all the possible genomic events.^107^ In parallel, ways to increase the efficiency and precision of this strategy are being developed, such as Cas nucleases with minimal collateral targeting,^108^, ^109^ Cas9 endowed with Myospreader sequences^110^ and guide RNAs engineered to improve the Cas9 performance and to achieve precise spatiotemporal control of CRISPR/Cas9.^111^ Improved delivery systems of the CRISPR/Cas9 components, as an alternative to AAV vectors, can also be considered. Different kind of lipid nanoparticles or self‐deliverable RNPs have been recently employed for transduction of CRISPR/Cas9 gene‐editing machinery^112^, ^113^, ^114^ and have the advantage of showing low immunogenicity compared with AAVs.^115^ Preclinical assessment of CRISPR/Cas9‐mediated‐phenotypic recovery in DMSXL mice is a crucial milestone to be reached before proceeding further and we have shown that this is feasible. In the next future it will be decisive to develop more efficient tools allowing to apply this therapy to humans. This is the first step encouraging the further development of a gene therapy potentially applicable to DM1 patients, alone or in combination with other therapies.

## AUTHOR CONTRIBUTIONS

*Conceptualisation*: Germana Falcone, Beatrice Cardinali and Fabio Martelli *Bioinformatics analysis*: Beatrice Cardinali, Claudia Provenzano, Jose Manuel Garcia‐Manteiga, Christine Voellenkle, and Spyros Tastsoglou *Funding acquisition*: Germana Falcone and Fabio Martelli *Investigation*: Mariapaola Izzo, Jonathan Battistini, Christine Voellenkle, Elisabetta Golini, Claudia Provenzano, Beatrice Cardinali, Denisa Baci and Silvia Mandillo *Methodology*: Tiziana Orsini, Georgios Strimpakos, Marcello Raspa, Germana Zaccagnini and Ferdinando Scavizzi *Project administration*: Germana Falcone and Fabio Martelli *Resources*: Genevieve Gourdon *Supervision*: Germana Falcone, Beatrice Cardinali and Fabio Martelli *Writing—original draft*: Germana Falcone *Writing—review and editing*: Fabio Martelli, Christine Voellenkle, Elisabetta Golini, Beatrice Cardinali, Claudia Provenzano and Silvia Mandillo. All authors read and approved the final manuscript.

## CONFLICT OF INTEREST STATEMENT

The authors declare no conflicts of interest.

## ETHICS STATEMENT

Animals were subjected to an experimental protocol approved by the Veterinary Department of the Italian Ministry of Health (no. 832/2019‐PR), and experiments were conducted according to the ethical and safety rules and guidelines for the use of animals in biomedical research provided by the relevant Italian laws and European Union's directives (no. 86/609/EEC and subsequent).

## Supporting information



Supporting Information

## Data Availability

The data that support the ONT findings of this study are available on GEO database (https://www.ncbi.nlm.nih.gov/geo, reference number GSE279054).
