# Supplementary material for: Muscle‐specific gene editing improves molecular and phenotypic defects in a mouse model of myotonic dystrophy type 1
Source: Clin Transl Med. 2025 Feb 16;15(2):e70227. doi: 10.1002/ctm2.70227 (PMC11830570; doi:10.1002/ctm2.70227)
Supplement: Supplementary file 1 — Supporting Information [file CTM2-15-e70227-s001.pdf]

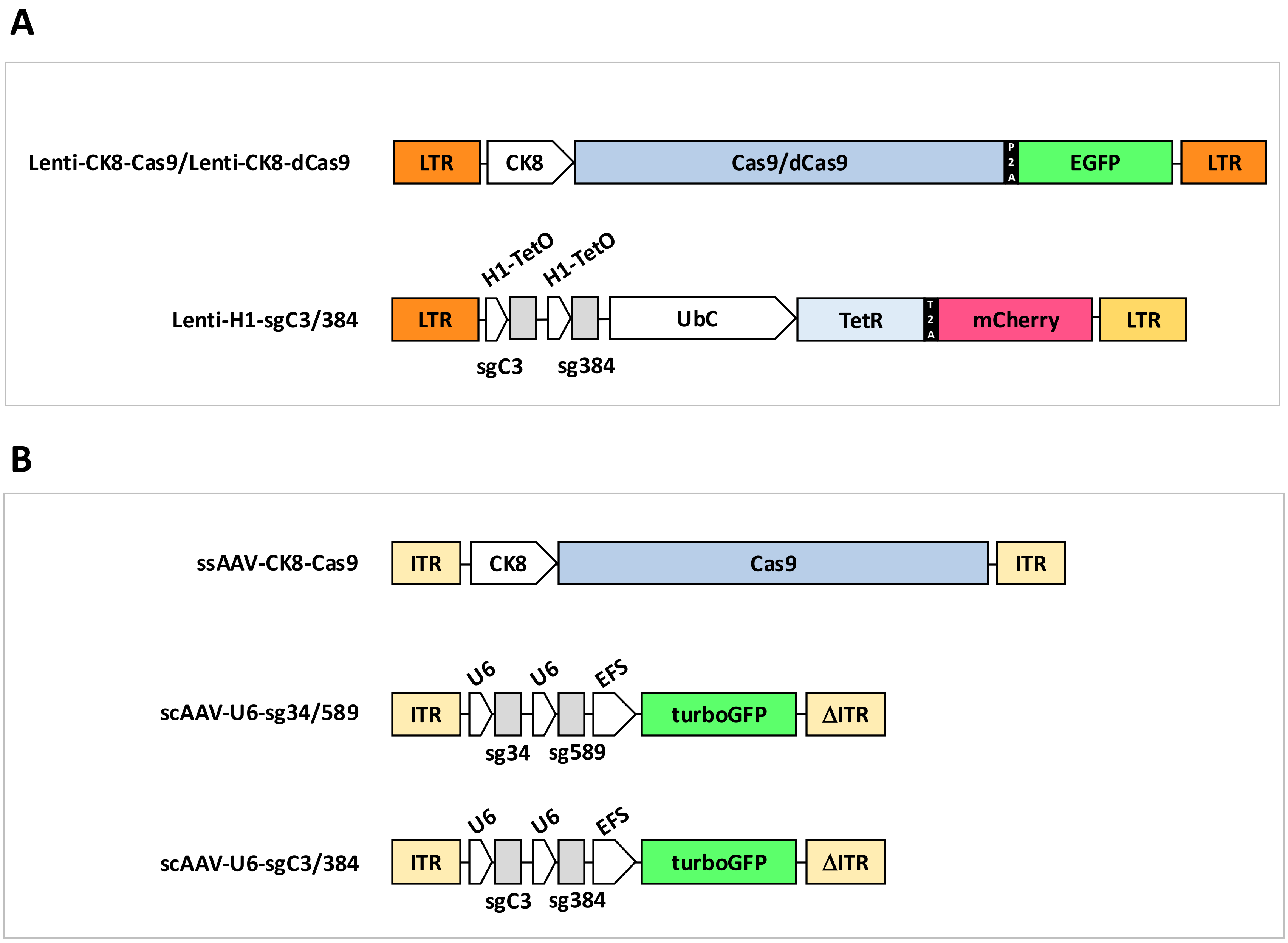

Figure S1. Viral vectors for CRISPR/Cas9 expression *in vitro* and *in vivo*

(A) Schematic representation of lentiviral constructs used for expression of either Cas9 or dCas9 and sgC3/384 in DM1 cells. (B) Schematic representation of AAV constructs used for expression of Cas9 and sg34/589 or sgC3/384 pairs in DMSXL mice. LTR, long terminal repeats; CK8, creatine kinase 8 promoter; Cas9/dCas9, eSpCas9 (1.1)/deSpCas9 (1.1) from *Streptococcus pyogenes;* P2A/T2A, 2A ribosomal skipping sequence; EGFP, enhanced green fluorescent protein; H1, H1 promoter; TetO, tetracycline operator; UbC, ubiquitin C promoter; TetR, tetracyclin repressor; ssAAV, single-strand-AAV; scAAV, self-complementary AAV, ITR, inverted terminal repeat; ΔITR, deleted inverted terminal repeat; U6, U6 human promoter; EFS, short elongation factor 1 promoter; turboGFP, turbo green fluorescent protein.

A

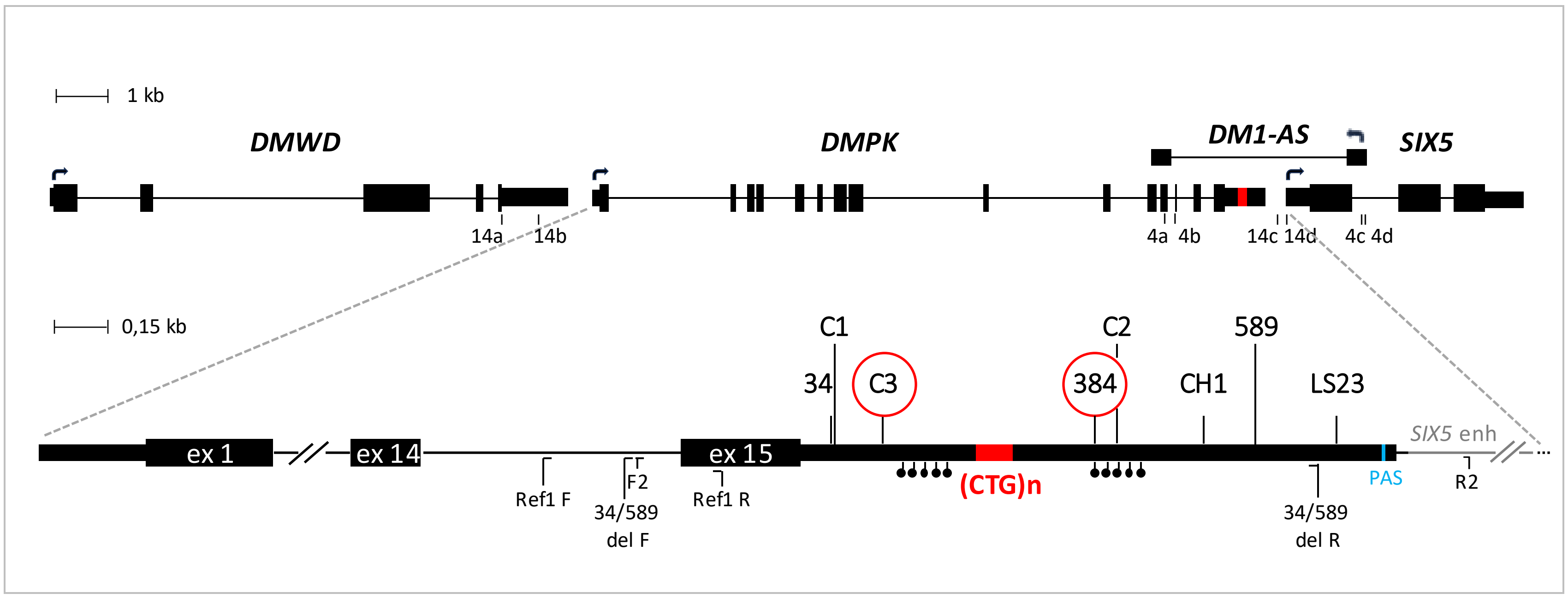

B

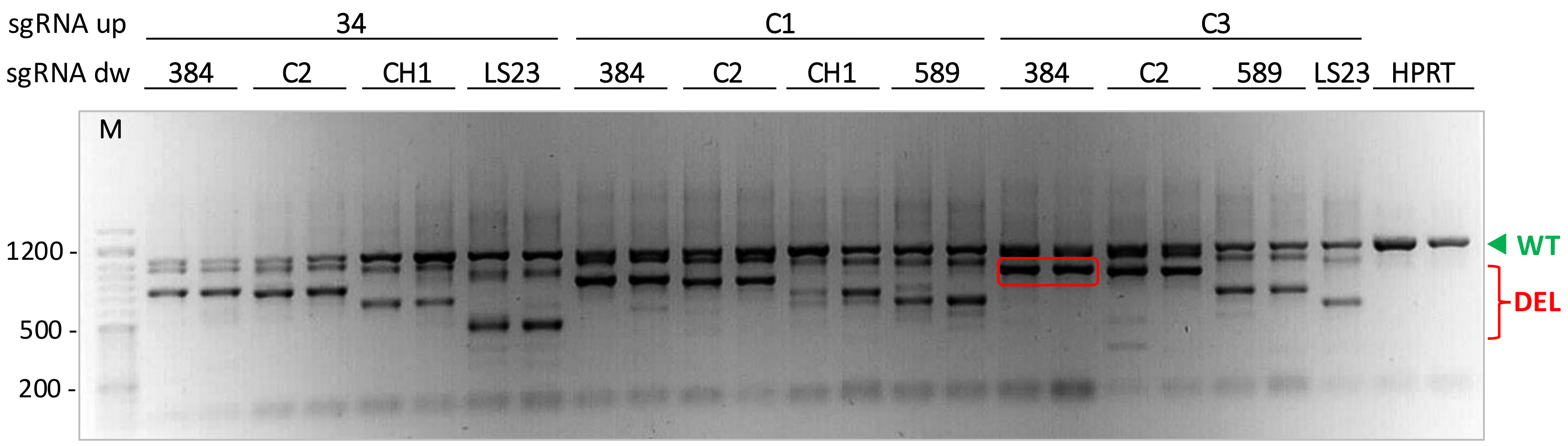

% Foci-negative nuclei													
34+384	34+C2	34+CH1	34+LS23	C1+384	C1+C2	C1+CH1	C1+589	C3+384	C3+C2	C3+589	C3+LS23	34+589	HPRT
ND	25-35	ND	20-25	25-35	20-25	ND	20-25	>40	20-25	20-25	ND	25-35	<10
	+++		++	+++	++		++	++++	++	++		+++	+

Figure S2. Design and test of sgRNAs targeting the CTG repeat region
(A) Schematic representation of the DMPK locus and surrounding genes (chr19:45,764,785..45,792,845), adapted from UCSC Genome Browser-hg38 assembly. Oxford Nanopore Technology crRNA binding sites are indicated (14a:Down.1696; 14b:Down.993; 4a:Down.11345; 4b:Down.1212; 14c:up227; 14d:up.365; 4c:up.2161; 4d:up.2270). CrRNA sequences are listed in Table S3A. The *DMPK* diagram illustrates the genomic binding sites for the designed sgRNAs, located both upstream (34, C1, C3) and downstream (384, C2, CH1, 589, LS23) of the CTG repeats, polyadenylation site (PAS), and the SIX5 enhancer region. The selected pair of sgRNAs (C3, 384) is highlighted in red. Black lollipops correspond to CTCF binding sites in the region surrounding the CTG repeats. Primers used for amplifying the deleted region (F2, R2; 34/589 del F, 34/589 del R) and for quantifying total DMPK copies (Ref1 F/Ref1 R) are shown. The sequences of the indicated sgRNAs and primers are listed in Table S1. (B) PCR analysis of genomic DNA from DM1 cells co-transfected with SpCas9 protein and *in vitro* transcribed sgRNA pairs (1 up and 1 down of CTG repeats) in all possible combinations. An sgRNA targeting the HPRT gene (HPRT) was used as negative control. The position of the amplicons corresponding to the wild type allele (WT) and the expected CTG-deleted products (DEL) are indicated in green and in red, respectively. Amplicons deriving from the selected pair of sgRNAs (C3, 384) are highlighted in red. The bottom table shows the percentage of foci-negative nuclei detected in DM1 cells transfected with the different sgRNA pairs. +: <10; ++: 20-25; +++: 25-35; ++++: >40; ND: not determined.

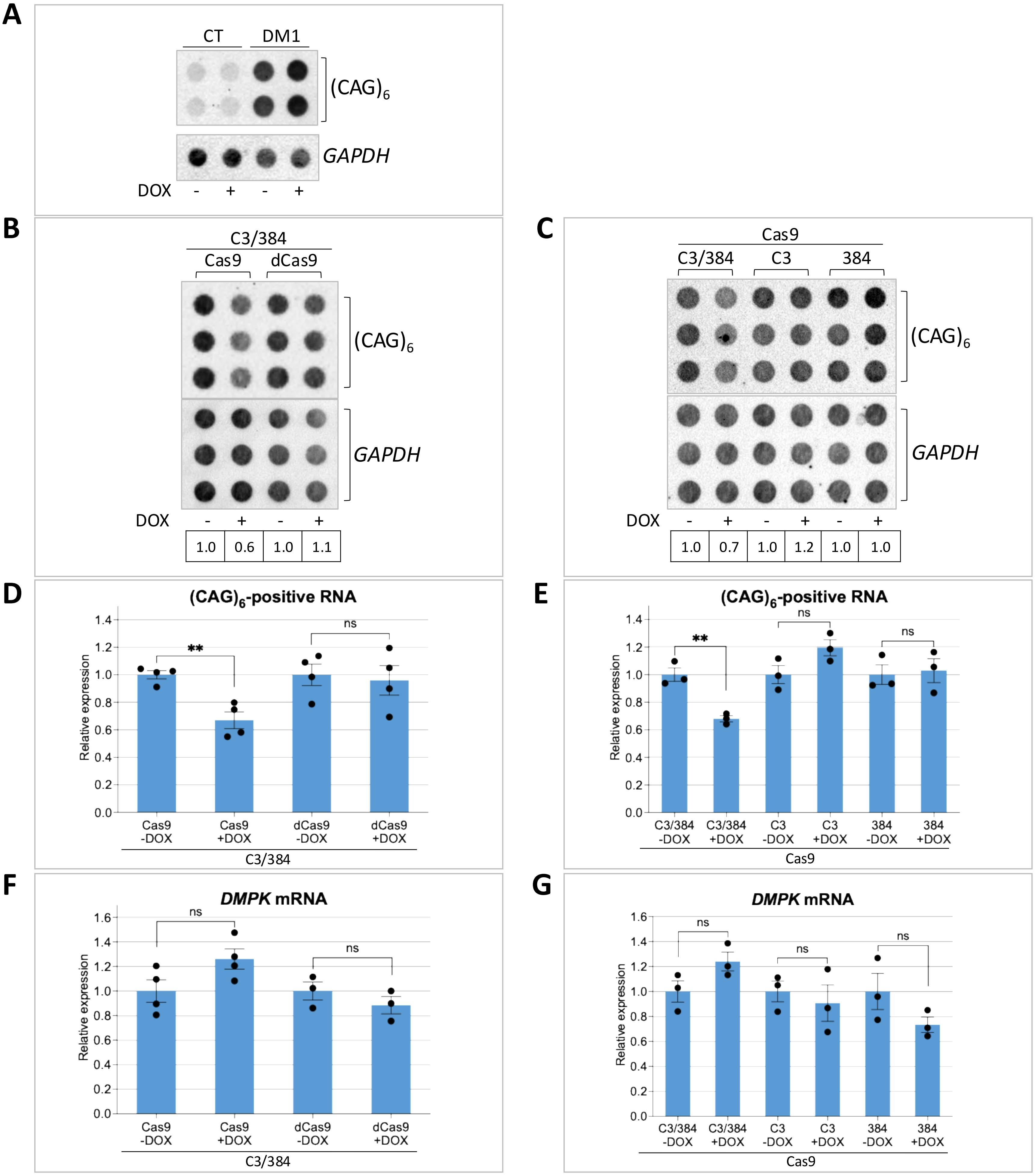

Figure S3. *DMPK* transcript expression in DM1 cells expressing Cas9/dCas9 and pair/single sgRNAs

(A) Dot-blot analysis of total RNA from control (CT) cells or DM1 cells, hybridized with $(CAG)_6$ and *GAPDH* probes. (B,D,F) DM1 cells transduced with lentiviruses expressing Cas9 or dCas9 and inducible sgC3/384 pair were treated with DOX for 5 days in GM. (B) Representative dot-blots of total or polyA$^+$ RNA hybridized as in (A). (D) Quantification of CUG-RNA expression in dot-blot experiments performed as in (B), normalized to *GAPDH* mRNA and expressed relative to untreated cells, set as 1 (mean ± SEM), n = 4. **p = 0.0028. (F) qRT-PCR analysis of total *DMPK* mRNA from the same samples analyzed in (D), normalized to *GAPDH* mRNA and expressed relative to untreated cells, set as 1 (mean ± SEM). (C,E,G) DM1 cells transduced with lentiviruses expressing Cas9 and inducible sgC3/384 pair or single sgRNAs were treated with DOX for 5 days in GM. (C) Representative dot-blots of total RNA hybridized as in (A). (E) Quantification of CUG-RNA expression in dot-blot experiments performed as in (C), normalized to *GAPDH* mRNA and expressed relative to untreated cells, set as 1 (mean ± SEM), n = 3. **p = 0.0039. (G) qRT-PCR analysis of total *DMPK* mRNA from the same samples analyzed in (E), normalized to *GAPDH* mRNA and expressed relative to untreated cells, set as 1 (mean ± SEM). The numbers below the dot-blots show quantification of CUG-mRNA, normalized to *GAPDH* mRNA, relative to untreated cells, set as 1, in the representative experiments shown. Each dot represents an individual sample from independent experiments. The statistical analyses presented in the figure were conducted using an unpaired t-test, with Welch's correction applied where appropriate. ns = not significant.

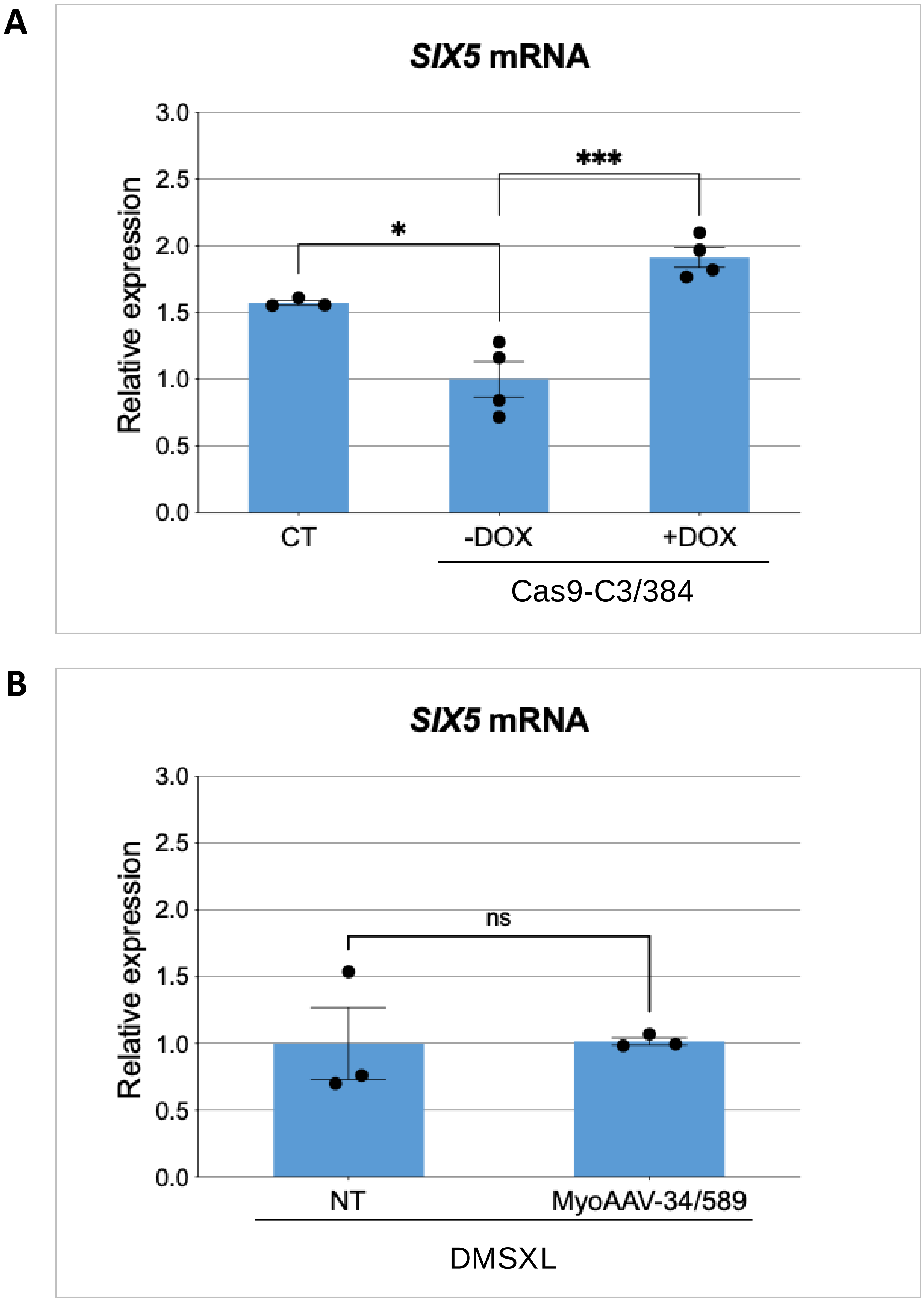

Figure S4. *SIX5* transcript expression following CTG-editing in DM1 cells and in homozygous DMSXL mice
(A) qRT-PCR analysis of *SIX5* mRNA expressed in control cells (CT) and in DM1 cells transduced with lentiviruses carrying Cas9 and DOX-inducible sgC3/384 pair, untreated or treated with DOX for 5 days in GM. Expression of *SIX5* transcript was normalized to *GAPDH* mRNA and expressed relative to the levels measured in DOX untreated cells set as 1 (mean ± SEM). Error bars indicate the standard error of the mean and each dot represents an individual sample from independent experiments. n = 3/4; *p = 0.0213, ***p = 0.00096. (B) qRT-PCR analysis of *SIX5* mRNA expressed in the heart of untreated (NT) and MyoAAV-34/589-treated homozygous DMSXL mice. Expression of *SIX5* transcript was normalized on *Gapdh* mRNA and expressed relative to the levels measured in untretead mice (NT) set as 1 (mean ± SEM). Error bars indicate the standard error of the mean and each dot represents a single individual. n = 3; ns = not significant. The statistical analyses presented in the figure were conducted using an unpaired t-test, with Welch's correction applied where appropriate.

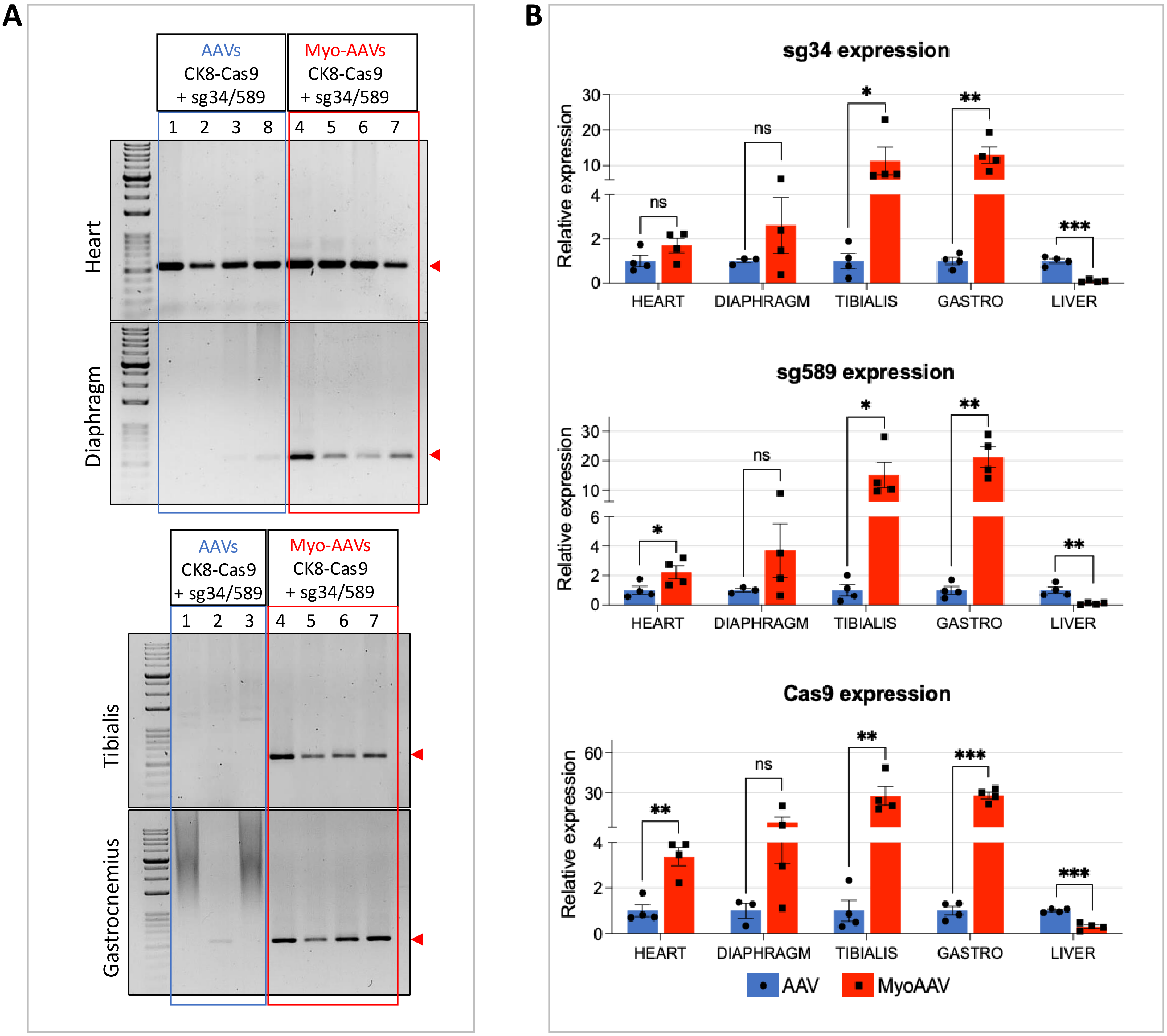

Figure S5. Comparative analysis of editing efficiency and expression of CRISPR/Cas9 components in mice injected with AAV9 and MyoAAV vectors. DMSXL hemizygous mice were injected at P5 with AAV9 (AAVs) and myotropic AAVs (MyoAAVs) transducing CK8-Cas9 and the sg34/589 pair. Four weeks later mice were sacrificed and heart, diaphragm, tibialis anterior (TA), gastrocnemius muscles, and liver (as a non-muscle tissue control) were analysed for gene editing (A) and for the expression of sg34, sg589 and Cas9 (B). n = 3-4; *p < 0.05, **p < 0.01, ***p < 0.001, ns = not significant. The red triangle indicates the expected CTG-deleted products. The statistical analyses presented in the figure were conducted using an unpaired t-test, with Welch's correction applied where appropriate.

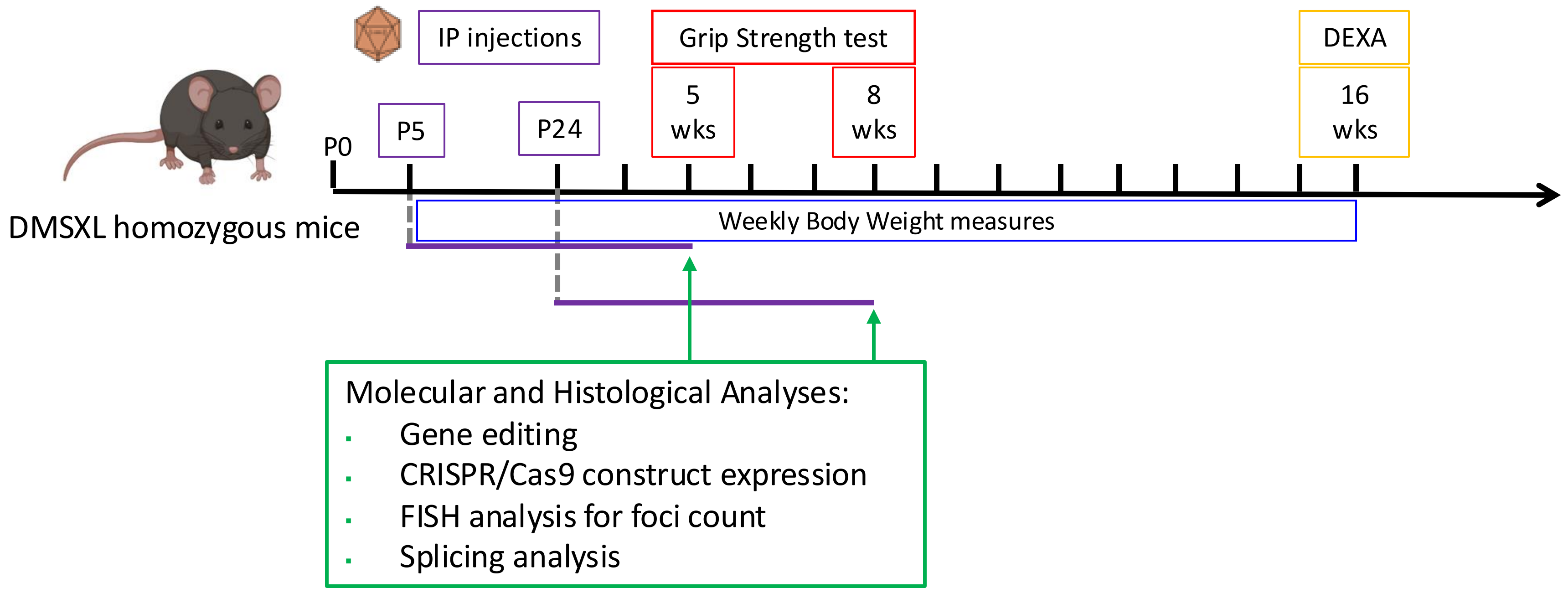

Figure S6. Timeline of *in vivo* experiments
Schematic representation of phenotypic assessment of mice treated with MyoAAV at P5 or P24 and their controls.

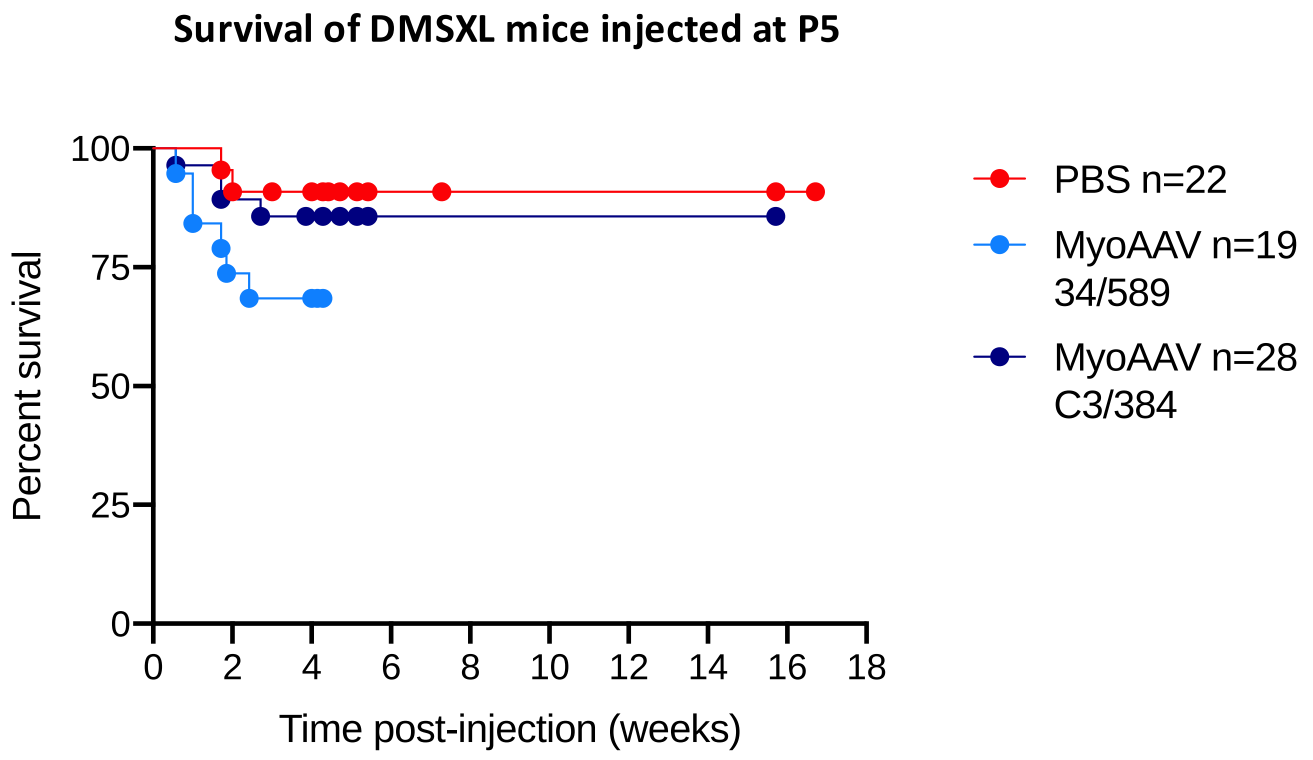

Figure S7. Survival of PBS and MyoAAV-treated DMSXL mice

Comparison of survival curves of mice injected at P5 with PBS (n = 22), MyoAAV-34/589 (n = 19) or MyoAAV-C3/384 (n = 28). Light blue line represents all mice injected with MyoAAV-34/589 and sacrificed four weeks post-injection. Six mice out of 19 (31.5%) died before sacrifice. For the other two groups, most of the mice were sacrificed four weeks after injection, the rest were sacrificed at different time points for other experiments (grip test, DEXA) up to 16/17 weeks post-injection. Of these, 2/22 PBS injected (9%) and 4/28 MyoAAV-C3/384 injected (14.2%) mice died before four weeks after injection. The survival curves are not significantly different: Log-rank (Mantel-Cox) test, Chi square 4.260, df 2, P = 0.118

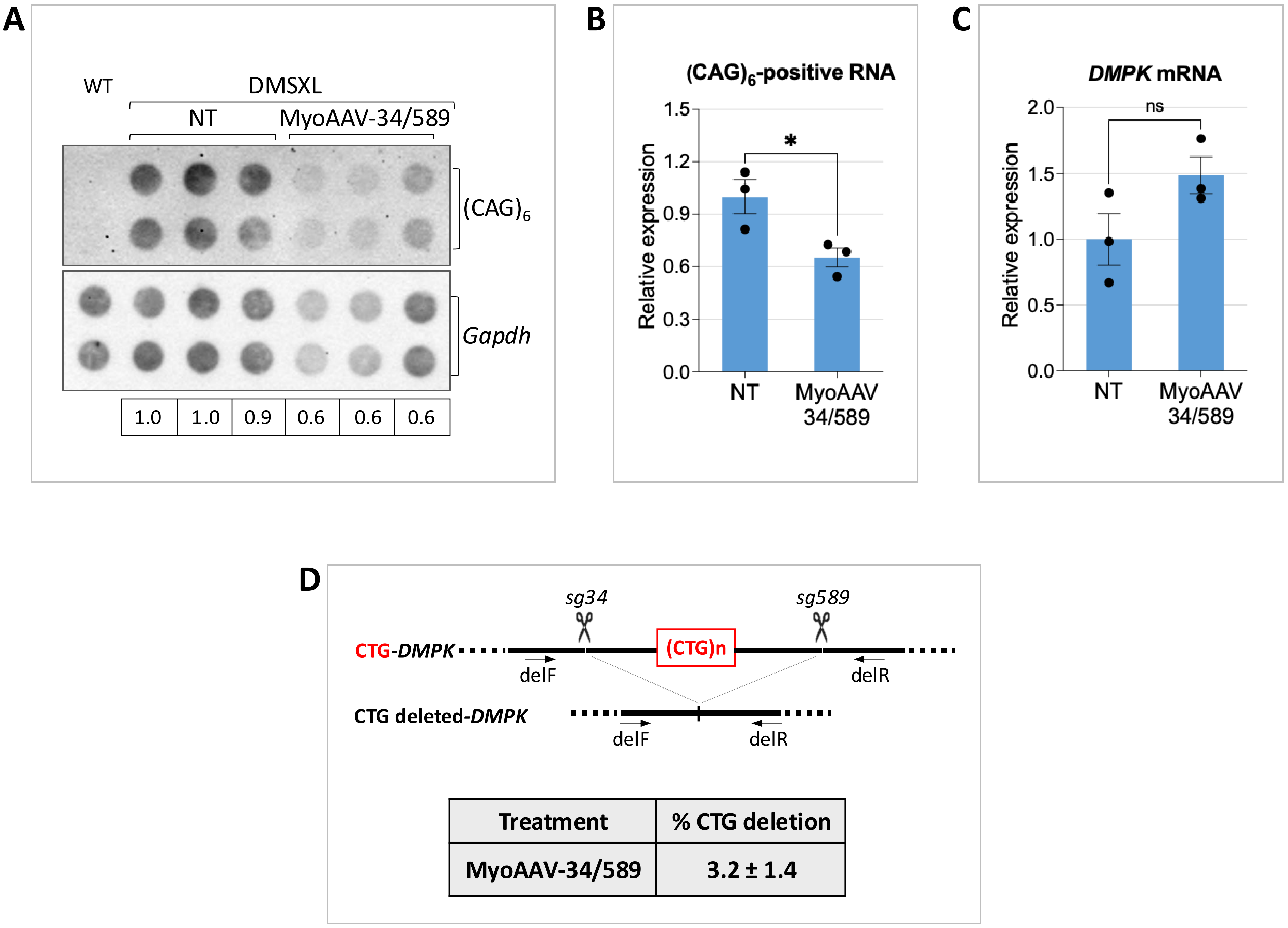

Figure S8. *DMPK* transcript expression and CTG-deletion efficiency in the heart of MyoAAV-34/589 treated DMSXL mice
(A) Dot-blot analysis of total RNA extracted from the heart of untreated (NT) and MyoAAV-34/589-treated homozygous DMSXL mice. Total RNA was hybridized with $(CAG)_6$ and *Gapdh* probes. Among the MyoAAV-treated mice, three showing a pronounced reduction in the number of foci were selected for the analysis (mice injected at P24). The numbers in the table show the level of CUG-mRNA expression normalized to *Gapdh* expression for each mice, relative to the first untreated mice set as 1, in a representative experiment. (B) Quantification of CUG-mRNA expression in the same mice as in (A), normalized to *Gapdh* expression, relative to untreated animals, set as 1, from three independent dot-blot experiments (mean ± SEM). n = 3; *p = 0.0356 (C) qRT-PCR analysis of total *DMPK* mRNA from the same mice as in (A), normalized to *Gapdh* mRNA and expressed relative to untreated animals, set as 1 (mean ± SEM). n = 3; ns = not significant. Error bars indicate the standard error of the mean and each dot represents a single individual. (D) qPCR analysis of CTG-repeat deletion in the heart of the same DMSXL mice described in (A). The percentage of deletion is calculated as described in Materials and Methods. (mean ± SEM); n = 3. The statistical analyses presented in the figure were conducted using an unpaired t-test, with Welch's correction applied where appropriate.

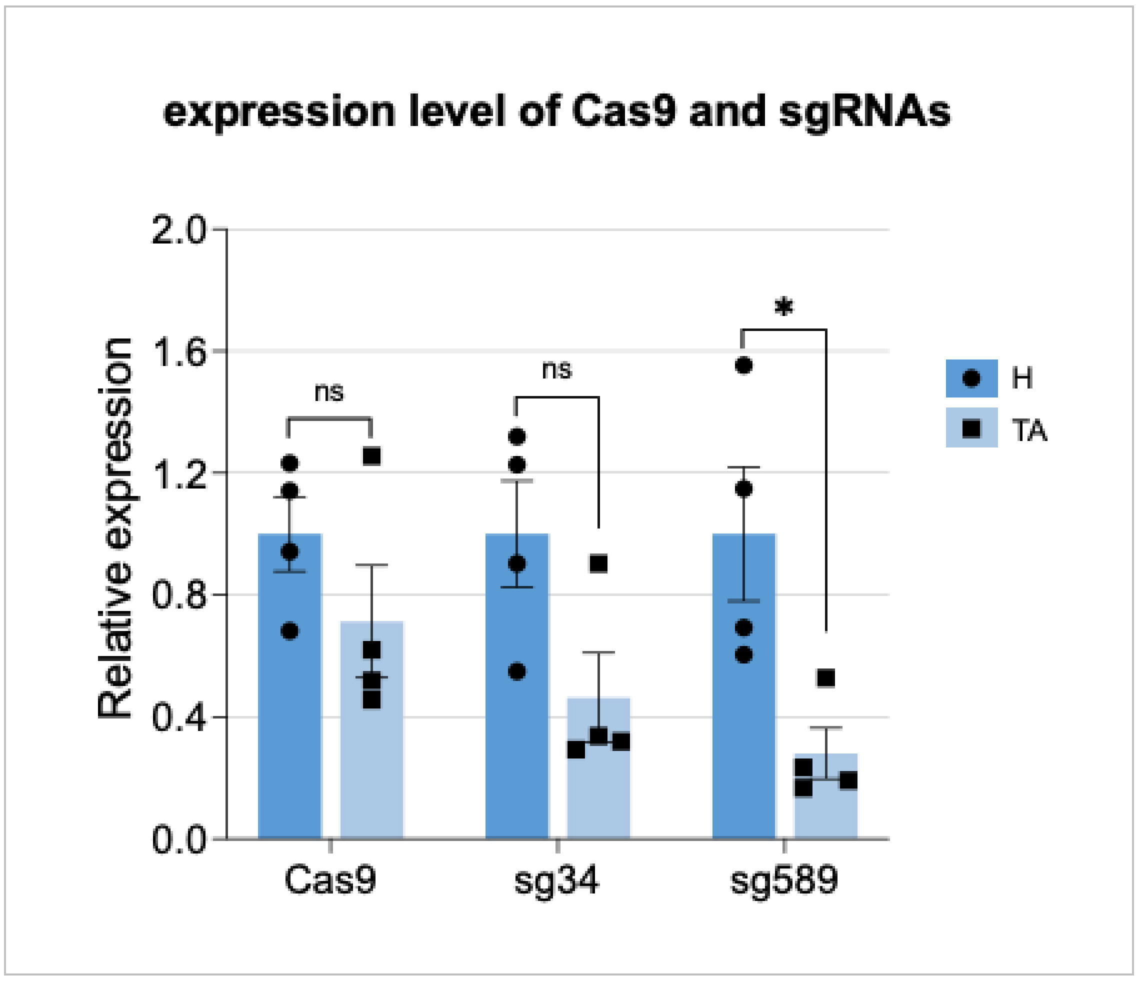

Figure S9. Comparative expression of CRISPR/Cas9 components in the heart and TA muscles of DMSXL mice injected with MyoAAVs

qRT-PCR analysis of Cas9, sg34 and sg589 expression in the heart and TA muscles of DMSXL mice systemically injected with MyoAAV-sg34/589 as described in Figure 3, normalized to *Rer1* mRNA expression, and shown in TA muscles relative to the heart, set as 1 (mean ± SEM). Expression of the two sgRNAs was analyzed using sgRNA-specific primers. Error bars indicate the standard error of the mean and each dot represents a single individual; $n = 4$; $^{*}p < 0.0222$, ns = not significant. The statistical analyses presented in the figure were conducted using an unpaired t-test, with Welch's correction applied where appropriate.

Body weight at age 1-16 weeks

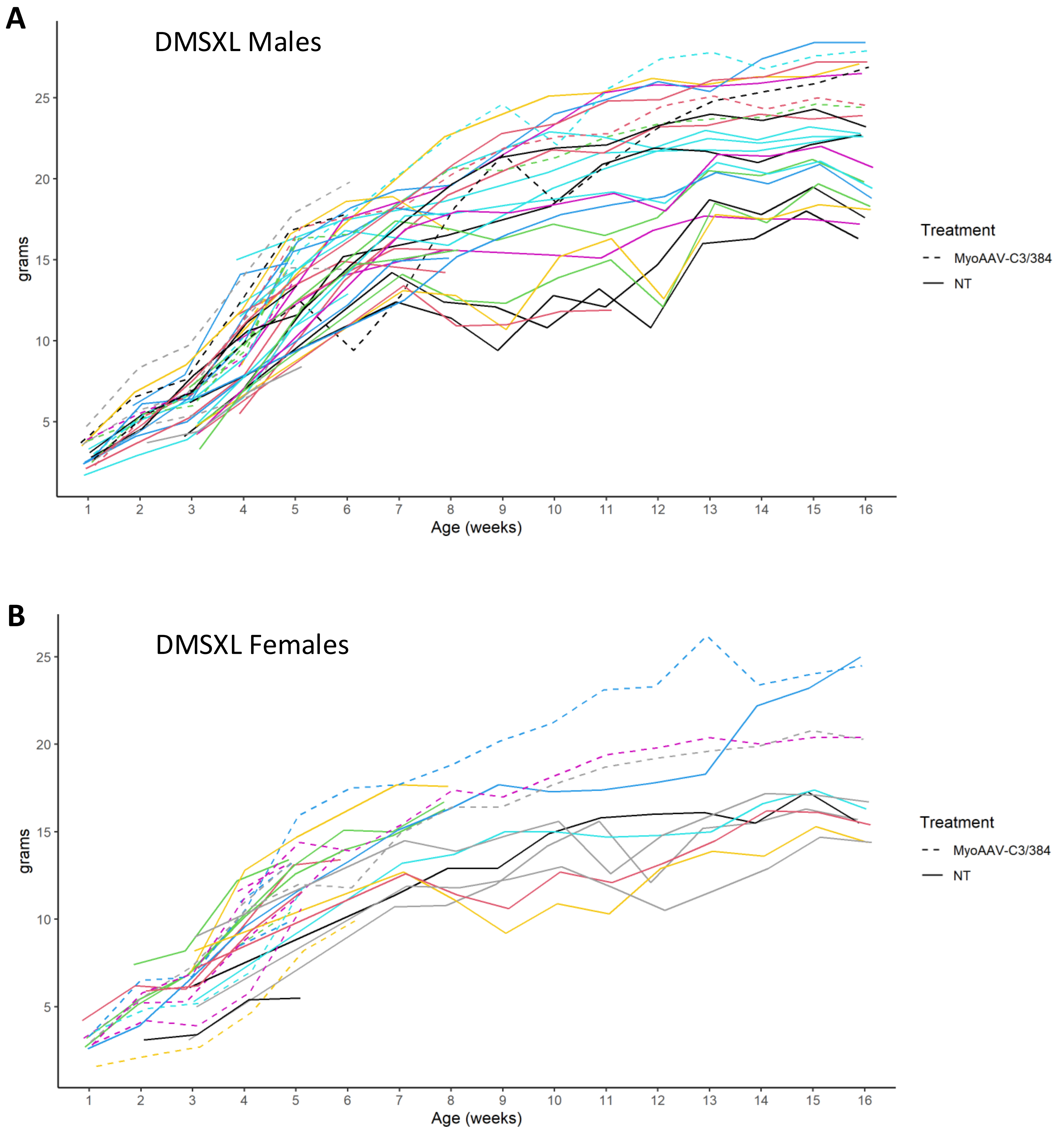

Figure S10. Body weight curves of MyoAAV-C3/384 treated DMSXL mice at age P5

Body weight curves of male (A) and female (B) mice of untreated (NT) and MyoAAV-C3/384 treated groups from age 1 to 16 weeks. The rate of weight gain significantly differs between NT and MyoAAV-C3/384 groups in males ($p = 8.25 \times 10^{-7}$) and females ($p = 4.38 \times 10^{-6}$), with NT exhibiting reduced gain in both sexes. Each line represents a single mouse, solid lines correspond to mice of the NT group and dashed lines to mice of the MyoAAV-C3/384 group. Not all mice reached the time point of 16 weeks (Table S4). Weight gain difference across groups over time was assessed using mixed effects models, considering individual variability as a random effects variable.

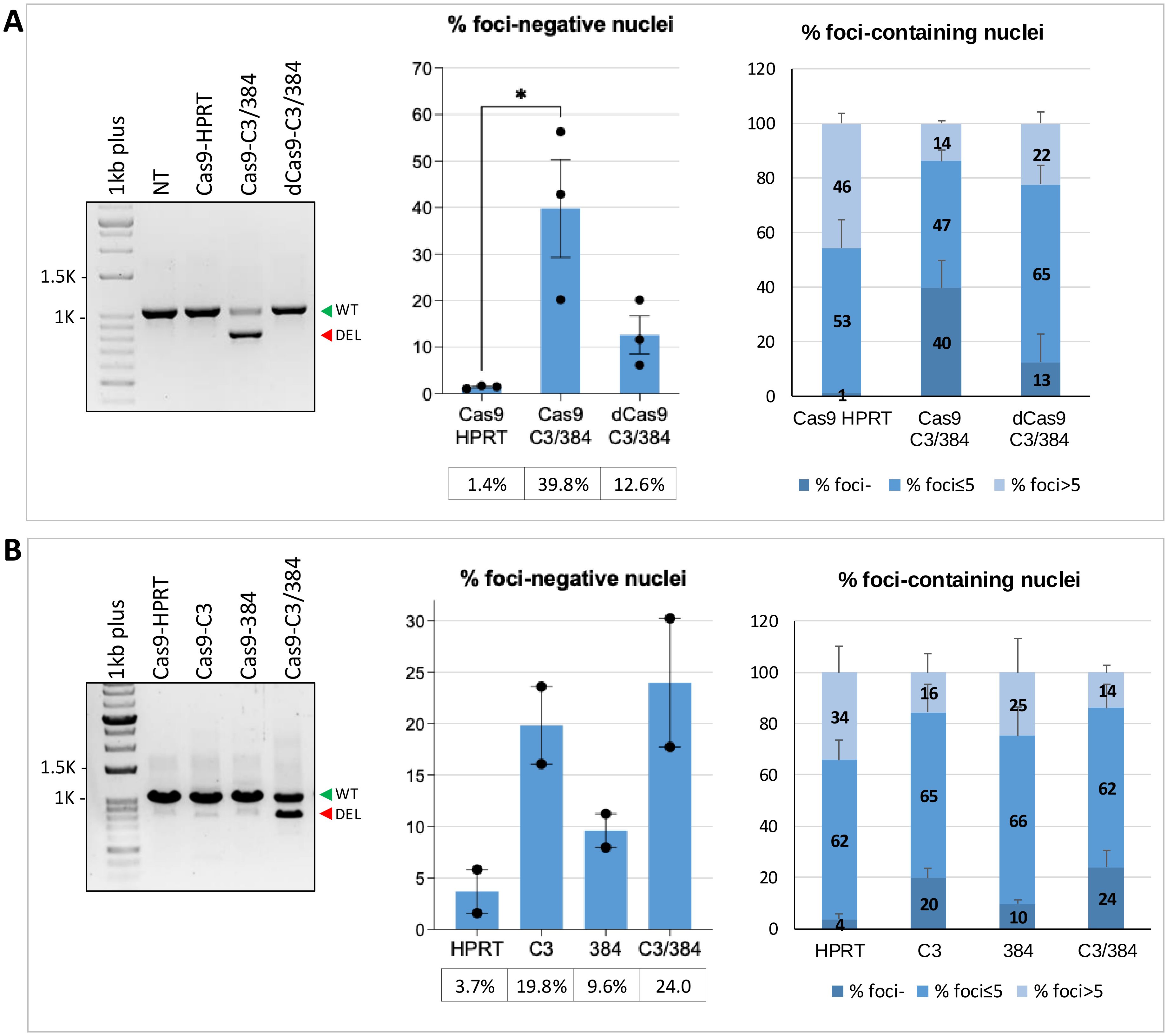

Figure S11. Ribonuclear foci decrease in DM1 cells transiently expressing dCas9 and single sgRNAs

DM1 cells were transiently transfected in GM with Cas9 or dCas9 protein and *in vitro* transcribed sgRNAs and, 2-days later, analyzed for *DMPK* gene editing and RNA FISH with a fluorescent $(CAG)_6CA$ probe. (A) Left: PCR analysis of *DMPK* gene editing (primers F2/R2) in non-transfected cells (NT) or cells transfected with Cas9 or dCas9 protein along with control HPRT-sgRNA and sgC3/384, as indicated. Middle: percentage of foci-negative nuclei measured in the transfected cells (mean ± SEM); *$p < 0.05$. Right: percentage of total nuclei containing no foci, ≤5 foci, and >5 foci.; n = 3. (B) Left: PCR analysis of *DMPK* gene editing in cells transfected with Cas9 protein along with control HPRT-sgRNA, sgC3, sg384 or sgC3/384, as indicated. Middle: percentage of foci negative nuclei measured in the transfected cells (mean ± SEM). Right: percentage of total nuclei containing no foci, ≤5 foci, and >5 foci. n = 2. Error bars indicate the standard error of the mean and each dot represents an individual sample from independent experiments. Green arrow = WT allele; red arrow = CTG-deleted alleles.

A

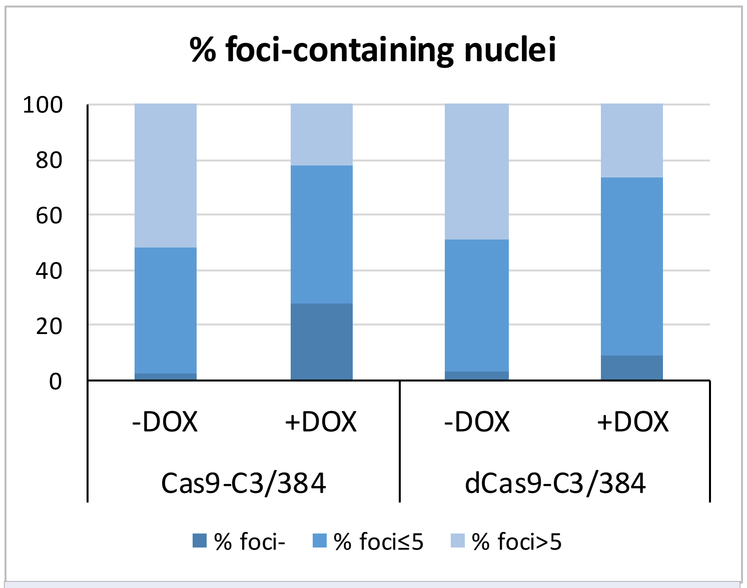

% of nuclei with/out foci in GM (n = 4)			
	% foci-	% foci≤5	% foci>5
Cas9-C3/384	2.5 ± 0.4	46.0 ± 4.7	51.5 ± 5.1
Cas9-C3/384 DOX	27.6 ± 2.9 (***)	50.5 ± 5.2	21.9 ± 2.8 (**)
dCas9-C3/384	3.2 ± 0.3	47.7 ± 2.8	49.1 ± 2.7
dCas9-C3/384 DOX	9.1 ± 0.6 (***)	64.6 ± 2.1 (**)	26.3 ± 2.6 (***)

B

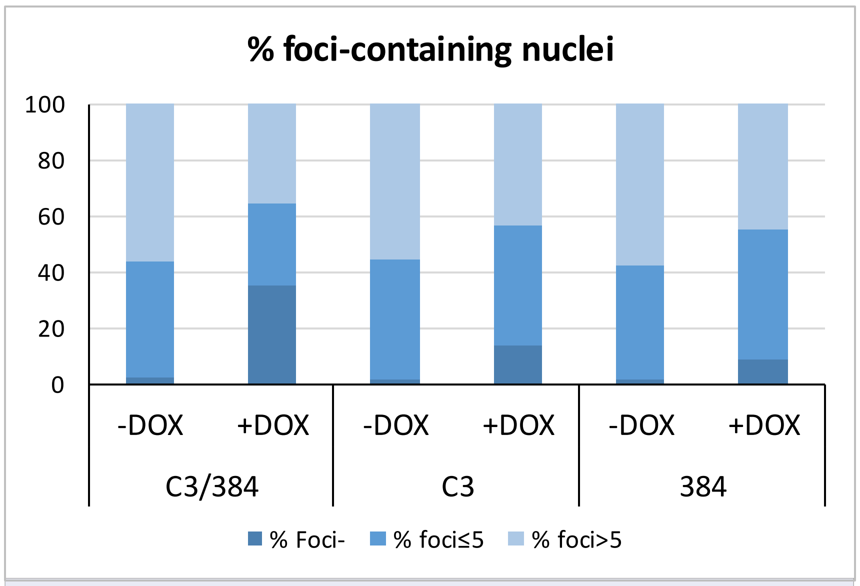

% of nuclei with/out foci in GM (n = 4)			
	% foci-	% foci≤5	% foci>5
C3/384	2.2 ± 0.5	41.4 ± 2.4	56.4 ± 2.4
C3/384 DOX	34.9 ± 2.8 (***)	29.6 ± 1.9 (**)	35.4 ± 2.5 (***)
C3	1.6 ± 0.2	43.0 ± 3.0	55.3 ± 3.0
C3 DOX	13.8 ± 0.2 (***)	43.0 ± 1.2	43.2 ± 1.2 (**)
384	1.5 ± 0.2	40.9 ± 3.1	57.6 ± 3.1
384 DOX	8.8 ± 0.9 (***)	46.6 ± 1.5	44.6 ± 1.5 (*)

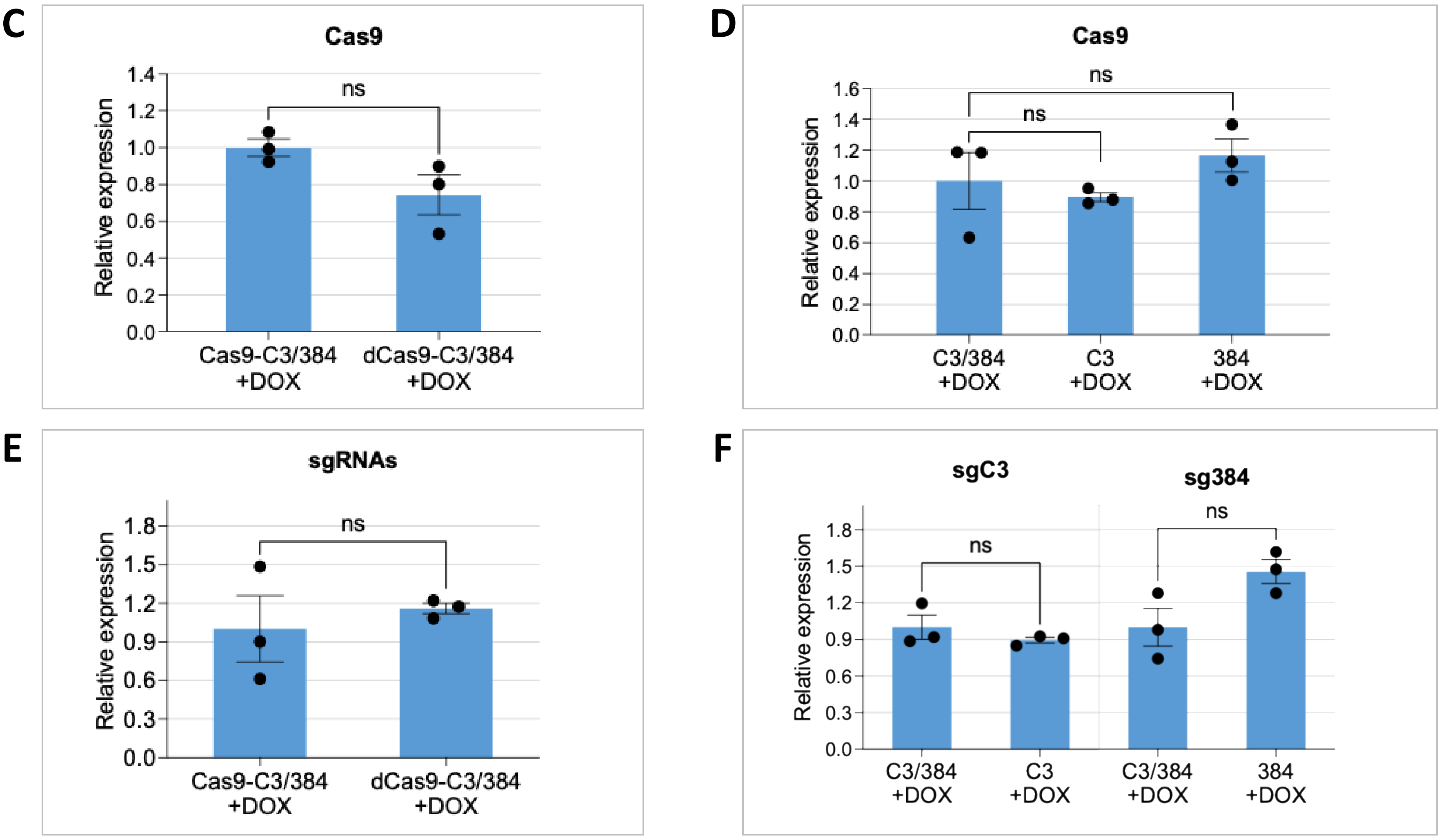

Figure S12. Decrease of nuclear foci in DM1 cells stably expressing dCas9 complexes or single sgRNA-Cas9 complexes
DM1 cells transduced with lentiviruses expressing Cas9 or dCas9 along with lentiviruses expressing inducible sgC3/384 pair (A,C,E) or with lentiviruses expressing Cas9 along with inducible sgC3/384 pair or single sgRNAs (B,D,F) were treated with DOX for 5 days in GM. (A) and (B) RNA FISH analysis of DM1 cells transduced as described above. Histograms show the percentage of total nuclei containing no foci, ≤5 foci, and >5 foci; **p < 0.01; ***p < 0.005; n = 4. (C-F) qRT-PCR expression analysis of Cas9, dCas9 and sgRNA transcripts in cells treated as described above. The primer pairs used for sgRNA analysis amplify both sgRNAs (E) or are specific for each sgRNA (F). Expression of Cas9/dCas9 and sgRNAs was normalized on *RPL23* mRNA and expressed relative to the levels measured in untreated cells set as 1 (mean ± SEM); n = 3; ns = not significant. Error bars indicate the standard error of the mean and each dot represents an individual sample from independent experiments. The statistical analyses presented in the figure were conducted using an unpaired t-test, with Welch's correction applied where appropriate.

Table S1. List of sgRNA target sequences and primers used for PCR analysis of genomic DNA and RNA

sgRNA Target Sequences	
sg34	GGGCACTCAGTCTTCCAACG
sgC1	GTTGGAAGACTGAGTGCCCG
sgC3	GCGGAGACCCACGCTCGGAG
sg384	GGTGCGTGGAGGATGGAACA
sgC2	GCACTTTGCGAACCAACGAT
sgCH1	GCGCGGGATCCCCGAAAAAG
sg589	AAATATCCAAACCGCCGAAG
sgLS23	AATAAATACCGAGGAATGT

PCR Primers			
DMPK F2	GTCCCAGGAGCCAATCAGAGG	DMPK R2	CTAGCTCCTCCCAGACCTTCG
sgRNAcom F	GTTTCAGAGCTATGCTGGAAAC	sgRNAcom R	CGACTCGGTGCCACTTTT
sg34RNA F	GGCACTCAGTCTTCCAACG	sgRNAcom R	CGACTCGGTGCCACTTTT
sg589RNA F	ATATCCAAACCGCCGAAG	sgRNAcom R	CGACTCGGTGCCACTTTT
sgC3RNA F	CGGAGACCCACGCTCGGAG	sgRNAcom R	CGACTCGGTGCCACTTTT
sg384RNA F	GTGCGTGGAGGATGGAACA	sgRNAcom R	CGACTCGGTGCCACTTTT
Cas9 F	CGGCACAGCATCAAGAAGAA	Cas9 R	TCTTCTGGCGGTTCTCTTCA
RPL23 F	TCCGGATTTCCTTGGGTCTT	RPL23 R	TGTTCAGCCGTCCCTTGATC
GAPDH F	CACCATCTTCCAGGAGCGAG	GAPDH R	CCTTCTCCATGGTGGTGAAGAC
SERCA incl 22 F	CAGTGGCTCATGGTCCTCAA	SERCA incl 22 R	GGGGAACAGTTATCCCTCTAGGT
SERCA excl 22 F	GGAACTACCTAGAGGATCCAGAAGA	SERCA excl 22 R	AGCTCTGCCTGAAGATGTGTC
SERCA com F	GGTGATCCGCCAGCTAATGA	SERCA com R	GGGCACCCTTGACAAACATC
INSR incl 11 F	CGAATGCTGCTCCTGTCCAA	INSR incl 11 R	GCCTGAAGAGGTTTTTCTGGG
INSR excl 11 F	TTTTCGTCCCCAGGCCATC	INSR excl 11 R	ACCAGCGACTCCTTGTTCAC
INSR com F	ATCGACTGGTCCCGTATCCT	INSR com R	AGATGGTCGGGCAAACTTTCT
INSR ex11 F	CCAAAGACAGACTCTCAGAT	INSR ex11 R	AACATCGCCAAGGGACCTGC
Ldb3 incl 9 F	TGGAGAATCCGAGGCCACA	Ldb3 incl 9 R	CTCGCTGTAGCTGGTATGGG
Ldb3 excl 9 F	TGCGAAGGTCAAGGCCACAG	Ldb3 excl 9 R	TGGTAGACAGAAGGCCGGAT
Ldb3 com F	GATGGCGTCAACACAGACAC	Ldb3 com R	AATAGGCCGCTTGGACTTCTG
Rer1 F	TTGATGGAAGACTCAGATGACGG	Rer1 R	AATGCCTTTTGTAGCTGCGTG
34/589 del F	GCGTAGGCGGGGCGAGTC	34/589 del R	CAGTGCATCCAAAACGTG
DMPK ref1 F	ATGACGAGTTCGGACGGGAT	DMPK ref1 R	CGAGACAGAACAACGGCGAA
DMPK mRNA F	AGATCGTCCACTACAAGGAGCA	DMPK mRNA R	GAAGGGATGTGTCCGGAAGT
SIX5 F	TGGTGGTGCTGGGGGTTGTATC	SIX5 R	GGGGCAGGGTGTTCCGCTTAC

Table S2A. Primers used for amplicon generation used for the off-target analysis by amplicon deep sequencing

Off-targets predicted for sgC3	illumina adapters	TARGET-SPECIFIC SEQUENCE
ND.1_F	tcgtcggcagcgtcagatgtgtataagagacag	AAAATTGCCGACGTGGCTTG
ND.1_R	gtctcgtgggctcggagatgtgtataagagacag	TTGTGTGAGCACTCCTTCCC
ND.2_F	tcgtcggcagcgtcagatgtgtataagagacag	AAAAATGTCAGGAGGGGCATGT
ND.2_R	gtctcgtgggctcggagatgtgtataagagacag	TGTGGACTCCAGACCTTTCTCTA
HGFAC_F	tcgtcggcagcgtcagatgtgtataagagacag	AGCACACTTACTGTCGGTGG
HGFAC.rev1_R	gtctcgtgggctcggagatgtgtataagagacag	CTGGCTTGAGAGAACTCGGG
ND.3_F	tcgtcggcagcgtcagatgtgtataagagacag	ACGGAGGTGTGGAGGAAGAG
ND.3_R	gtctcgtgggctcggagatgtgtataagagacag	CCGTGAGAAATTGAGCACAGC
ENSG00000287100_F	tcgtcggcagcgtcagatgtgtataagagacag	CACTGCTGCACTGTGGGAG
ENSG00000287100_R	gtctcgtgggctcggagatgtgtataagagacag	GCTGCTTGCCCAGGTACTA
ENSG00000259124_F	tcgtcggcagcgtcagatgtgtataagagacag	GAGGATGCTAATGGGCAGCA
ENSG00000259124_R	gtctcgtgggctcggagatgtgtataagagacag	TGCCTACTATGCACCCAGTC
LRMDA_F	tcgtcggcagcgtcagatgtgtataagagacag	ATTACAGGCATGCACCACCA
LRMDA_R	gtctcgtgggctcggagatgtgtataagagacag	AGCTGGACAACGGATTTGGA
ALOX12-AS1_F	tcgtcggcagcgtcagatgtgtataagagacag	GGCCACGGAATTATCTGCCT
ALOX12-AS1_R	gtctcgtgggctcggagatgtgtataagagacag	TCGAGCTCCTGTTCCCGATA
ND.6_F	tcgtcggcagcgtcagatgtgtataagagacag	GCCCCATTTGAGGAGCACTT
ND.6_R	gtctcgtgggctcggagatgtgtataagagacag	GGTGCTAAACCCCTCACTGC
RefSeq_LOC107985856_F	tcgtcggcagcgtcagatgtgtataagagacag	CCTCCATGCACCTGGGAAG
RefSeq_LOC107985856_R	gtctcgtgggctcggagatgtgtataagagacag	CTCCATAACTGGAGATTAAGACCT
ABCG1_F	tcgtcggcagcgtcagatgtgtataagagacag	AGTGGAGCTCCTTCCATCAG
ABCG1_R	gtctcgtgggctcggagatgtgtataagagacag	GCATTCACTCCTGTTCTCTCCA
EPHB2_F	tcgtcggcagcgtcagatgtgtataagagacag	TCTAGACCCTTCAACTTGCCA
EPHB2_R	gtctcgtgggctcggagatgtgtataagagacag	ACACCTTCAAGTCAGGCTCC
HGFAC_F	tcgtcggcagcgtcagatgtgtataagagacag	TCCTGAGTCTCCGAGATGCT
HGFAC_R	gtctcgtgggctcggagatgtgtataagagacag	GCCTTTGGTGCCTAGTGTCA
ND.8_F	tcgtcggcagcgtcagatgtgtataagagacag	GGTATCAGTAGCAACCCCCG
ND.8_R	gtctcgtgggctcggagatgtgtataagagacag	CAGAAGCCAGCTTTGCCATC
SLC38A10_F	tcgtcggcagcgtcagatgtgtataagagacag	AACACCAAGTGTGTCTCAGGT
SLC38A10_R	gtctcgtgggctcggagatgtgtataagagacag	GGAAAGTCAGTCTGTGTGCG
ENSG00000285663_F	tcgtcggcagcgtcagatgtgtataagagacag	GGGACCGCAGGTATGTTTCC
ENSG00000285663_R	gtctcgtgggctcggagatgtgtataagagacag	CCAGACAGGATGGGTAGCCT
ENSG00000289079_F	tcgtcggcagcgtcagatgtgtataagagacag	CTCCTACTTTGGCCGCACTT
ENSG00000289079_R	gtctcgtgggctcggagatgtgtataagagacag	AGGCCCGGTGAGAAATTGAG
LRP5_F	tcgtcggcagcgtcagatgtgtataagagacag	CTACGCACACGTCCCTTTCA
LRP5_R	gtctcgtgggctcggagatgtgtataagagacag	GGCATCTGCAGTAGTCGGAG
ND.10_F	tcgtcggcagcgtcagatgtgtataagagacag	CAAGGCTAGCAAAGGCTCAG
ND.10_R	gtctcgtgggctcggagatgtgtataagagacag	CATTCCACTGGGGCCAAGG
SPECC1_F	tcgtcggcagcgtcagatgtgtataagagacag	CTCTGCTTCTCCTGCTTCTCG
SPECC1_R	gtctcgtgggctcggagatgtgtataagagacag	GGACCAAGTAGGATGGAAATATGA
ND.11_F	tcgtcggcagcgtcagatgtgtataagagacag	CAGCTCCCTCTGCTTGTGG
ND.11_R	gtctcgtgggctcggagatgtgtataagagacag	AATTCAAGCATGGTGCAGGC

Off-targets predicted for sg384	illumina adapters	TARGET-SPECIFIC SEQUENCE
CEP104_F	tcgtcggcagcgtcagatgtgtataagagacag	TCATGTCAGTGAAGTGCTGCT
CEP104_R	gtctcgtgggctcggagatgtgtataagagacag	TCTCTAGACCAAGCGGGTGA
PDE1C_F	tcgtcggcagcgtcagatgtgtataagagacag	ACCAGGTAGCTAAGTCTGTGG
PDE1C_R	gtctcgtgggctcggagatgtgtataagagacag	CCCTGTGTGCCAGATGTTC
ND.1_F	tcgtcggcagcgtcagatgtgtataagagacag	ACACAATGCCCACAACAGGA
ND.1_R	gtctcgtgggctcggagatgtgtataagagacag	AGCAGCATTTATGTGCGCTG
NRXN3_F	tcgtcggcagcgtcagatgtgtataagagacag	ACTCAGTTTGGGACAGGGCT
NRXN3_R	gtctcgtgggctcggagatgtgtataagagacag	TCCATGAACAACTGGGCCTAC
CBY3_F	tcgtcggcagcgtcagatgtgtataagagacag	GAGGATCAGGCCCTGGGATA
CBY3_R	gtctcgtgggctcggagatgtgtataagagacag	GAGGTTGCAGTGAGCCAAGA
SLC2A5_F	tcgtcggcagcgtcagatgtgtataagagacag	CAGCTTTGGGTACGTACTGGG
SLC2A5_R	gtctcgtgggctcggagatgtgtataagagacag	CAGACATCTGGGGACCGTGT
Gene C7orf50_F	tcgtcggcagcgtcagatgtgtataagagacag	CCACAGGCTCCCAGTGTCA
Gene C7orf50_R	gtctcgtgggctcggagatgtgtataagagacag	TTCTGGCAGTAGGTTTCAGGT
ND.3_F	tcgtcggcagcgtcagatgtgtataagagacag	ACACTCACACTGATCTGCCG
ND.3_R	gtctcgtgggctcggagatgtgtataagagacag	ACATTGGAGACGTTGCCTGT
ZFP42.a_F	tcgtcggcagcgtcagatgtgtataagagacag	CCCTGTAATGGTTTCCTTGGGA
ZFP42.a_R	gtctcgtgggctcggagatgtgtataagagacag	TGAATGTCTGGAAAAGGCCCC
ZFP42.b_F	tcgtcggcagcgtcagatgtgtataagagacag	GTCCGGCCCATCTTCTAACC
ZFP42.b_R	gtctcgtgggctcggagatgtgtataagagacag	GTGGGAAGAGACGAGCGAAG
ULBP1_F	tcgtcggcagcgtcagatgtgtataagagacag	GCCAGGGAACGTGGATAGAAA
ULBP1_R	gtctcgtgggctcggagatgtgtataagagacag	TTTCGGAACCTCTCCCACAAAT
LRFN2_F	tcgtcggcagcgtcagatgtgtataagagacag	GCTGGCCCTTGTATCCCTTTG
LRFN2_R	gtctcgtgggctcggagatgtgtataagagacag	GGCCTCCAGAACGTAGCATC
PARD3_F	tcgtcggcagcgtcagatgtgtataagagacag	GTGGGAACAGCTCAGTAGGT
PARD3_R	gtctcgtgggctcggagatgtgtataagagacag	CCTGGGACCTTAGGTGTTCAT
HDAC4_F	tcgtcggcagcgtcagatgtgtataagagacag	AACGTCCCACAATCCCAAAGT
HDAC4_R	gtctcgtgggctcggagatgtgtataagagacag	CATGCAGTGCTCTAAGGGTG
ND.4_F	tcgtcggcagcgtcagatgtgtataagagacag	GGGCGGTTTATGAAAAAGGGG
ND.4_R	gtctcgtgggctcggagatgtgtataagagacag	AAGAACCAGTGAGCCTGAGT
ENSG00000286563_F	tcgtcggcagcgtcagatgtgtataagagacag	TCCCTGACTCTTCATTGCCCA
ENSG00000286563_R	gtctcgtgggctcggagatgtgtataagagacag	CTCAAACTAAGTGCAGCCAGC
ND.5_F	tcgtcggcagcgtcagatgtgtataagagacag	AGGGCAAGTTGTGAGATGGG
ND.5_R	gtctcgtgggctcggagatgtgtataagagacag	TCATGGGTGTTGAGGTGTCC
AKR1C8_F	tcgtcggcagcgtcagatgtgtataagagacag	TCCACACCAGCTTGTCCTTG
AKR1C8_R	gtctcgtgggctcggagatgtgtataagagacag	TCAAGAGTTTCAGGAGGTGGT
ND.9_F	tcgtcggcagcgtcagatgtgtataagagacag	GTTCGTCAGACGTTTGCATGG
ND.9_R	gtctcgtgggctcggagatgtgtataagagacag	CTCCAAGCCTTCTCCAAGCC
SEMA5B_F	tcgtcggcagcgtcagatgtgtataagagacag	AAAATGACCTGCCCCAAGACT
SEMA5B_R	gtctcgtgggctcggagatgtgtataagagacag	GGCAGTGGAAATCAGGTCAGT
ND.11_F	tcgtcggcagcgtcagatgtgtataagagacag	GACACTGCCATATGTGCCCC
ND.11_R	gtctcgtgggctcggagatgtgtataagagacag	CGAGCTGGCTTTGCAATAGGA
TECPR2_F	tcgtcggcagcgtcagatgtgtataagagacag	CTGCCCACCCTTCACAACC
TECPR2_R	gtctcgtgggctcggagatgtgtataagagacag	ATTGGGAAGCTCTGTCATGCT

Table S2B. Investigation of sequence variations in potential sgC3 and sg384 off-targets.

Potential Targets of sgC3	Amplicon (genomic location)				Variant type (RefSNP ID)	Alternate allele frequency similar in Ctrl and Edited	DEPTH OF COVERAGE								
							DM_Edited			GM_Edited			DM_Not.Edited		
	Strand	Chr	Start	End			1	2	3	1	2	3	1	2	3
ND.1 (a)	+	chr17	77794365	77794537	none		6458	5372	9782	9514	24734	9240	12535	19607	13249
ND.2	+	chr12	5169663	5169962	none		49916	31519	127597	77064	85052	21016	123562	163769	52347
HGFAC	+	chr4	3448417	3448627	none		92842	150563	67150	62490	88213	84995	105398	60568	16901
ND.3	+	chr5	106246240	106246428	none		1275	500	997	515	329	215	1021	1095	851
ENSG00000287100	+	chr6	119440922	119441153	none		2081	1356	2901	1313	1891	2913	1472	1737	1535
ENSG00000259124	+	chr14	76509007	76509285	none		125483	150434	45176	46533	135278	116320	188190	174319	101486
LRMDA	+	chr10	76201233	76201518	none		65145	35366	53566	63972	92555	144408	43180	109082	116659
ALOX12-AS1	+	chr17	6894486	6894773	none		77513	31647	37049	82137	14067	47293	104857	83000	167446
ND.6	+	chr13	75518449	75518692	none		242	244	201	113	193	111	281	186	75
LOC107985856	+	chr2	20479824	20479930	none		18663	4170	9523	4812	7158	1187	589	9035	20433
ABCG1	+	chr21	42271248	42271513	none		82132	82430	42185	30717	64261	84240	68927	118549	252263
EPHB2	+	chr1	22813146	22813351	none		136522	104157	89754	69646	111552	32068	96358	109528	600335
HGFAC	+	chr4	3448327	3448599	none		95368	31476	179157	36622	151943	211622	101453	180603	210834
SLC38A10	-	chr17	81287315	81287603	none		79469	69214	69836	37614	50660	61547	57510	41989	394710
ENSG00000285663	-	chr15	102487143	102487435	none		11	16	13	5	6	2	57	13	2
ENSG00000289079	-	chr9	102487143	102487435	none		426834	177820	325565	359814	280243	191162	149983	353732	270300
LRP5	-	chr11	68351459	68351770	none		55639	112771	100400	79081	61479	41077	125561	52552	62002
ND.10	-	chr9	114121577	114121889	none		47912	99939	14338	80683	102184	45103	35545	207334	365231
SPECC1	-	chr17	20073412	20073693	none		287421	216759	351159	129298	13823	108048	134436	124234	84621
ND.11	-	chr8	92429403	92429642	rs57588467	yes	48626	67944	23891	7395	4949	17561	14500	7901	7548
					rs58097237	yes									
					rs7009528	yes									

Potential Targets of sg384	Amplicon (genomic location)				Variant type (RefSNP ID)	Alternate allele frequency similar in Ctrl and Edited	DEPTH OF COVERAGE								
							DM_Edited			GM_Edited			DM_Not.Edited		
	Strand	Chr	Start	End			1	2	3	1	2	3	1	2	3
CEP104	+	chr1	3846430	3846754	none		292856	144911	67091	26617	51272	80166	22357	12790	66032
PDE1C	+	chr7	32262262	32262549	none		117004	271816	251975	24465	15502	13544	31369	58162	230
ND.1	+	chr4	1553215	1553531	rs28530496	yes	229016	136395	97742	45638	27350	25785	14340	13027	52238
NRXN3	+	chr14	78530316	78530640	rs8006051	yes	121747	205171	52633	53944	52535		27652	22389	33304
SLC2A5	+	chr1	9038414	9038698	none		142537	108063	42177	35959	26465	67441	98999	57082	43828
GeneC7orf50	+	chr7	1101068	1101363	none		274566	312684	279305	109663	21347	65695	79929	43653	90075
ZFP42.a	+	chr4	187996872	187997187	rs1560911044	yes	91747	155433	77648	64627	135289	153914	128389	171187	66510
ZFP42.b	+	chr4	187996822	187997114	rs1560911044	yes	79349	116768	35460	123891	49808	195686	215182	94410	27518
ULBP1	+	chr6	149971019	149971283	none		28799	223853	230029	209588	279500	102106	195250	226270	136718
LRFN2	+	chr6	40482095	40482385	none		94768	106560	103302	218217	161680	165727	153802	115076	57960
PARD3	+	chr10	34641783	34642001	rs2496721	yes	170296	152727	291807	374920	127420	78988	449505	188475	309918
					rs1570650	yes	170296	152727	291807	374920	127420	78988	449505	188475	309918
HDAC4	+	chr2	239117694	239117958	none		136803	135805	177146	158537	89810	320467	219646	334786	317982
ND.4	+	chr13	105209610	105209878	none		322164	66891	64614	419663	272041	257644	221189	93707	367856
ENSG00000286563	-	chr12	67350398	67350692	none		90397	311890	339752	179251	302883	34487	130139	139840	97346
ND.5	-	chr2	104335921	104336209	none		144495	217539	66469	88795	79660	151171	144094	223733	362843
AKR1C8	-	chr10	5160974	5161257	rs7090038	yes	184829	139794	246091	248282	303260	178726	154498	43123	429561
ND.9	-	chr20	24800277	24800589	none		31908	16679	72243	74396	77962	89846	17736	74191	17131
SEMA5B	-	chr3	122968607	122968877	none		134670	207053	93261	103445	178255	303922	48125	181935	243621
ND.11	-	chr10	128927810	128928104	rs1037431	yes	85774	87412	299367	320706	225735	163215	157090	340671	75357
TECPR2	-	chr14	102492432	102492732	none		193029	94945	206104	121572	224476	112119	58748	161262	156726

[a] "Not described" (ND) refers to a genomic location that does not overlap with any annotated gene.

Table S2C. Alternate allele frequencies of observed variants in potential sgC3 and sg384 off-targets.

sgRNA	Potential Targets of sgRNA	Amplicon				Variant information	ALTERNATE ALLELE FREQUENCY								
							DM_Edited			GM_Edited			DM_Not.Edited		
		Strand	Chr	Start	End		1	2	3	1	2	3	1	2	3
sgC3	ND.11	-	chr8	92429403	92429642	rs57588467	0.44	0.55	0.44	0.48	0.46	0.52	0.46	0.40	0.49
						rs58097237	0.45	0.55	0.44	0.49	0.47	0.52	0.47	0.40	0.49
						rs7009528	0.45	0.55	0.44	0.49	0.47	0.52	0.47	0.40	0.49
sg384	ND.1	+	chr4	1553215	1553531	rs28530496	0.47	0.47	0.49	0.54	0.45	0.44	0.50	0.51	0.48
	NRXN3	+	chr14	78530316	78530640	rs8006051	0.47	0.52	0.51	0.51	0.51		0.51	0.51	0.49
	ZFP42.a	+	chr4	187996872	187997187	rs1560911044	0.70	0.63	0.66	0.66	0.65	0.65	0.69	0.72	0.65
	ZFP42.b	+	chr4	187996822	187997114	rs1560911044	0.69	0.63	0.66	0.65	0.65	0.64	0.69	0.72	0.65
	PARD3	+	chr10	34641783	34642001	rs2496721	0.49	0.49	0.52	0.50	0.50	0.49	0.49	0.50	0.48
						rs1570650	1.00	1.00	1.00	1.00	1.00	1.00	1.00	1.00	1.00
	AKR1C8	-	chr10	5160974	5161257	rs7090038	0.48	0.47	0.49	0.48	0.49	0.48	0.50	0.47	0.51
	ND.11	-	chr10	128927810	128928104	rs1037431	1.00	1.00	1.00	0.99	1.00	1.00	1.00	1.00	1.00

Table S3A. Guiding RNA sequences used for Cas9-mediated target enrichment for ONT.

cRNA_pool_14 kb	SEQUENCE + PAM
**UP.227**	CCCCTCTTCTCGACGCTCGG**TGG**
**UP.395**	TCGGAATTCCCGGCTCCGCA**GGG**
**Down.1696**	CAACTCCCCGAGTGGCACAG**TGG**
**Down.993**	GGACCTGCGAGTCACACAAC**TGG**
**cRNA_pool_4 kb**	**SEQUENCE + PAM**
**UP.2270**	GTGTGATTCCCCAACACCGA**TGG**
**UP.2162**	ACTTAGTCCCCGCGCCCCGC**GGG**
**Down.1212**	GGCTCGGAACCGGGACCTAG**AGG**
**Down.11345**	ACTTCGCCAGGTCGGGATCG**GGG**

Table S3B: Sequencing metrics of data-sets obtained by ONT.

Sample	Total Throughput (B=bases)	Bytes	Total Reads
GF5_GM	868 MB	1.6Gb	67.528
GF8_GM	655.8 MB	1.2Gb	38.332
GF2_DM	1.7 GB	3.1Gb	129.153
GF7_DM	329.6 MB	638.1Mb	29.233
GF9_DM	332.8 MB	644.7Mb	27.535

Table S3C. On-target analysis of sgRNA C3/384 editing outcome in proliferating (GM) and differentiated (DM) cells carrying

Sample	Total reads	WT allele		Mutated DM1 allele		CTG excision		Inversion	
GM	140	87	62%	18	13%	33	24%	2	1%
DM	151	56	37%	34	23%	55	36%	6	4%

CTG EXCISION	GM				DM			
	Number of events		*Length [bp]*		*Number of events*		*Length [bp]*	
			Median	*Range*			*Median*	*Range*
Deletion	26	67%	8	(1-633)	14	23%	1	(1-383)
Insertion	9	23%	1	(1-3)	27	45%	1	(1-3)
Precise	4	10%			19	32%		

sgC3 TARGET REGION	GM				DM			
	Number of events		*Length [bp]*		*Number of events*		*Length [bp]*	
			Median	*Range*			*Median*	*Range*
Deletion	46	39%	6	(1-1883)	22	25%	2	(1-24)
Insertion	20	17%	1	(1-5)	24	27%	1	(1-3)
No change	52	44%			43	48%		

sg384 TARGET REGION	GM				DM			
	Number of events		*Length [bp]*		*Number of changes*		*Length [bp]*	
			Median	*Range*			*Median*	*Range*
Deletion	38	31%	8	(1-172)	23	25%	1	(1-65)
Insertion	39	32%	1	(1-5)	37	40%	1	(1-3)
No change	45	37%			33	35%		

Table S3D. On-target analysis of sgRNA C3/384 editing outcome in proliferating (GM) and differentiated (DM) cells carrying human heterozygous DM1; per sample.

Sample	Total reads	WT allele		Mutated DM1 allele		CTG excision		Inversion	
GF5_GM	37	23	62%	4	11%	9	24%	1	3%
GF8_GM	103	64	62%	14	14%	24	23%	1	1%
GF2_DM	107	31	29%	29	27%	42	39%	5	5%
GF7_DM	22	13	59%	1	5%	8	36%	0	0%
GF9_DM	22	12	55%	4	18%	5	23%	1	5%

CTG EXCISION	GF5_GM				GF8_GM				GF2_DM				GF7_DM				GF9_DM			
	Number of events		*Length [bp]*		Number of events		*Length [bp]*		*Number of events*		*Length [bp]*		*Number of events*		*Length [bp]*		*Number of events*		*Length [bp]*	
			Median	*Range*			*Median*	*Range*			*Median*	*Range*			*Median*	*Range*			*Median*	*Range*
**Deletion**	11	(73%)	6	(1-389)	15	(63%)	49	(1-633)	10	(24%)	1	(1-384)	2	(15%)	1	(1-1)	2	(50%)	1	(1-1)
**Insertion**	3	(20%)	1	(1-2)	6	(25%)	1	(1-3)	17	(40%)	1	(1-3)	7	(54%)	1	(1-5)	3	(50%)	1	(1-2)
**Precise**	1	(7%)			3	(12%)			15	(36%)			4	(31%)			0	0%		

sgC3 TARGET REGION	GF5_GM				GF8_GM				GF2_DM				GF7_DM				GF9_DM			
	Number of events		*Length [bp]*		*Number of events*		*Length [bp]*		*Number of events*		*Length [bp]*		*Number of events*		*Length [bp]*		*Number of events*		*Length [bp]*	
			Median	*Range*			*Median*	*Range*			*Median*	*Range*			*Median*	*Range*			*Median*	*Range*
**Deletion**	10	(37%)	19	(1-176)	36	(40%)	3	(1-1883)	16	(28%)	2	(1-24)	3	(20%)	15	(1-24)	3	(19%)	1	(1-1)
**Insertion**	5	(19%)	1	(1-1)	15	(16%)	1	(1-5)	17	(29%)	1	(1-3)	4	(27%)	1	(1-1)	3	(19%)	1	(1-1)
**No change**	12	(44%)			40	(44%)			25	(43%)			8	(53%)			10	(62%)		

sg384 TARGET REGION	GF5_GM				GF8_GM				GF2_DM				GF7_DM				GF9_DM			
	Number of events		*Length [bp]*		*Number of events*		*Length [bp]*		*Number of events*		*Length [bp]*		*Number of events*		*Length [bp]*		*Number of events*		*Length [bp]*	
			Median	*Range*			*Median*	*Range*			*Median*	*Range*			*Median*	*Range*			*Median*	*Range*
**Deletion**	11	(35%)	3	(1-11)	27	(29%)	11	(1-172)	15	(25%)	2	(1-34)	4	(25%)	6	(1-7)	4	(24%)	1	(1-65)
**Insertion**	11	(35%)	1	(1-2)	28	(31%)	1	(1-5)	27	(45%)	1	(1-3)	6	(37.5%)	1	(1-4)	4	(24%)	1	(1-4)
**No change**	9	(30%)			36	(40%)			18	(30%)			6	(37.5%)			9	(52%)		

Table S4. **Body weight age 1 to 16 weeks**

	WT Males			NT Males			MyoAAV-C3/384 Males			WT Females			NT Females			MyoAAV-C3/384 Females		
**Age (weeks)**	**Mean**	**SEM**	**N**	**Mean**	**SEM**	**N**	**Mean**	**SEM**	**N**	**Mean**	**SEM**	**N**	**Mean**	**SEM**	**N**	**Mean**	**SEM**	**N**
1	N/A	N/A	N/A	2.66	0.172	10	3.29	0.242	10	N/A	N/A	N/A	3,175	0.366	4	2,914	0.241	7
2	N/A	N/A	N/A	4.8	0.33	12	5.76	0.313	10	N/A	N/A	N/A	5,286	0.544	7	4.9	0.526	7
3	9,767	0.308	12	5,736	0.319	22	6.96	0.353	10	10,950	0.564	12	6.32	0.424	15	5,429	0.639	7
4	15,922	0.690	46	8,317	0.594	30	10,392	0.564	12	14,453	0.595	34	7,807	0.641	15	9,108	0.666	12
5	19,868	0.364	19	13,077	0.694	13	15,075	0.535	12	18,058	0.297	19	11.95	0.987	8	12	0.614	12
6	24,194	0.495	18	15,535	0.448	17	16,243	1,297	7	19,700	0.933	4	14.4	0.552	5	13.42	1,269	5
7	24,623	0.312	13	15,835	0.56	17	17.45	1,618	4	19,942	0.358	12	13.6	0.613	11	16	0.862	3
8	27,345	0.425	33	16,352	0.571	25	20,525	0.941	4	21,733	0.555	18	13,864	0.752	11	17,533	0.696	3
9	26,815	0.403	13	15,858	1,351	12	22,125	0.876	4	21,258	0.445	12	13,062	0.954	8	17,867	1,179	3
10	29,629	0.609	21	18,284	0.941	19	21,125	0.915	4	21,592	0.400	12	14.2	0.698	8	19,033	1,093	3
11	31,162	0.773	21	19,011	0.978	19	23	0.956	4	22,600	0.638	12	13,787	0.862	8	20.4	13,656	3
12	31,838	1,024	21	19,467	1,109	18	24.65	0.956	4	22,317	0.498	12	14,012	0.818	8	20,767	1,278	3
13	34,024	0.781	21	21,422	0.687	18	25.35	0.87	4	24,067	0.564	12	15,088	0.674	8	22,067	2.08	3
14	34,881	0.782	21	21.25	0.778	18	25,075	0.665	4	24,683	0.486	12	16,213	0.998	8	21.1	1.15	3
15	35,695	0.825	21	22,128	0.722	18	25,775	0.666	4	25,725	0.645	12	17,175	0.925	8	21,733	1,139	3
16	35,663	1,345	19	21,456	0.843	18	25,925	0.876	4	23,310	0.457	10	16,675	1,223	8	21,733	1,384	3

Body weight in grams (mean, SEM, N) of male and female wild-type (WT), DMSXL homozygous untreated controls (NT) and DMSXL homozygous mice injected at P5 with MyoAAV-C3/384 sgRNA guides
